# How the future of the global forest sink depends on timber demand, forest management, and carbon policies

**DOI:** 10.1016/j.gloenvcha.2022.102582

**Published:** 2022-08

**Authors:** Adam Daigneault, Justin S. Baker, Jinggang Guo, Pekka Lauri, Alice Favero, Nicklas Forsell, Craig Johnston, Sara B. Ohrel, Brent Sohngen

**Affiliations:** a University of Maine, USA; b Louisiana State University, USA; c International Institute for Applied Systems Analysis, Austria; d Georgia Institute of Technology, USA; e Bank of Canada, Canada; f U.S. Environmental Protection Agency, USA; g Ohio State University, USA; h North Carolina State University, USA

**Keywords:** Model intercomparison, Land use, Carbon, Bioenergy, Climate change mitigation, Shared socioeconomic pathways, Shared policy analysis

## Abstract

Deforestation has contributed significantly to net greenhouse gas emissions, but slowing deforestation, regrowing forests and other ecosystem processes have made forests a net sink. Deforestation will still influence future carbon fluxes, but the role of forest growth through aging, management, and other silvicultural inputs on future carbon fluxes are critically important but not always recognized by bookkeeping and integrated assessment models. When projecting the future, it is vital to capture how management processes affect carbon storage in ecosystems and wood products. This study uses multiple global forest sector models to project forest carbon impacts across 81 shared socioeconomic (SSP) and climate mitigation pathway scenarios. We illustrate the importance of modeling management decisions in existing forests in response to changing demands for land resources, wood products and carbon. Although the models vary in key attributes, there is general agreement across a majority of scenarios that the global forest sector could remain a carbon sink in the future, sequestering 1.2–5.8 GtCO2e/yr over the next century. Carbon fluxes in the baseline scenarios that exclude climate mitigation policy ranged from −0.8 to 4.9 GtCO2e/yr, highlighting the strong influence of SSPs on forest sector model estimates. Improved forest management can jointly increase carbon stocks and harvests without expanding forest area, suggesting that carbon fluxes from managed forests systems deserve more careful consideration by the climate policy community.

## 1. Introduction

The global forest sector is widely recognized in the scientific and policy communities for its contribution to the global carbon cycle and climate change mitigation (IPCC, 2018; Lauri et al., 2017; Grassi et al., 2017; Roe et al., 2019; Canadell and Raupach, 2008; Friedlingstein et al., 2019; Domke et al., 2020). Natural climate solutions such as avoided deforestation (Kindermann et al., 2008), afforestation (Bastin et al., 2019; Busch et al., 2019), forest restoration (Lewis et al., 2019), and improved forest management (Griscom et al., 2017; Austin et al., 2020) are important components of climate change mitigation goals. Despite this noted importance, knowledge gaps regarding the combined impact of future socioeconomic, management, and policy change on forest carbon stocks and greenhouse gas (GHG) emissions remain (Forsell et al., 2016; Popp et al., 2017). Key gaps include the role of timber demand on carbon flux, the influence of climate change policies on forest management and timber production, and the regional variation in carbon and wood product harvest outcomes.

Global-scale terrestrial carbon storage analyses often use book-keeping methods that assign carbon density parameters to land cover types and track land use over time (Houghton and Nassikas, 2017) or project impacts from discrete land use change (LUC) decisions via integrated assessment models (IAM) (Popp et al., 2017; Roe et al., 2019) that often assume all global forests are unmanaged or hold forest attributes (e.g., carbon sequestration rates) constant over time. Using LUC as the primary driver of forest dynamics ignores a critical component of the terrestrial carbon cycle – carbon storage in existing forests – which is affected by harvesting, management interventions, and natural disturbance (Law et al., 2018). In addition, the IAM representation of the forest sector can be highly aggregated and include limited forest types (e.g., tropical natural) and carbon pools (e.g., aboveground). As a result, IAMs typically fail to account for the importance of region-specific management of existing forests and investment in new forestland, which is driven by a mix of socioeconomic change, market dynamics, land use policy, and interactions between pulpwood, sawtimber, and bioenergy demand systems. Further, the influence of forest management and investment are absent from bookkeeping and dynamic global vegetation models. Historical assessments of forest area and carbon flux are useful for identifying where impacts occur, but they often do not recognize the significance of accounting for socioeconomic and policy drivers behind these impacts (Harris et al., 2021). Market and management dynamics are important when modeling land use and carbon (Tian et al., 2018), especially for complex forest ecosystems that jointly produce raw materials for commodity markets and carbon sequestration to support climate goals.

This paper presents results from a first of its kind global forest model inter-comparison project (ForMIP) to estimate future forest area, carbon, harvests, and market outcomes across harmonized scenarios using three detailed economic models of the global forest sector – the Global Timber Model (GTM), Global Biosphere Management Model (GLO-BIOM), and Global Forest Products Model (GFPM). This study contributes to a rich literature of model inter-comparison exercises in the climate domain, including the Energy Modeling Forum (EMF) (Weyant et al., 2006; Fawcett et al., 2018), the Agricultural Model Comparison Project (AgMIP) (Nelson et al., 2014; Valin et al., 2014), and the Land Use Model Inter-comparison Project (LUMIP) (Lawrence et al., 2016; Ito et al., 2020). Our focus on the inter-comparison of forest sector models (FSM) is critical given the sector’s outsized influence on the global carbon cycle relative to its contribution to the global economy, as well as its recognized importance as a potential source of mitigation (Kindermann et al., 2008). In particular, FSMs reflect heterogeneity in the forest resource base, ecological constraints, management opportunities, product markets, and land use and management responses to market and environmental change (Lauri et al., 2017; Favero et al., 2018; Johnston and Radeloff, 2019; Nepal et al., 2019b, 2019a; Hu, Iordan and Cherubini, 2018; Daigneault, 2019; Favero, Daigneault and Sohngen, 2020; Gomes et al., 2020; Jones et al., 2019; Estoque et al., 2019; Eriksson et al., 2020; Daigneault and Favero, 2021; Latta, Sjølie and Solberg, 2013).

We model future socioeconomic and climate policy change across three FSMs and 81 pathways through 2105 using the Shared Socioeconomic Pathways (SSP) (O’Neill et al., 2014, 2017; Daigneault et al., 2019), Representative Concentration Pathways (RCP) (O’Neill et al., 2017; Popp et al., 2017), and Shared Policy Assumptions (SPA) (O’Neill et al., 2020) approach commonly applied by IAMs. We add to the literature by a) harmonizing SSP-RCP-SPA assumptions in FSMs (O’Neill et al., 2017; Daigneault et al., 2019) and b) illustrating how incorporating a more detailed representation of the forest sector can capture forest ecosystem, market, and carbon dynamics not always accounted for in bookkeeping and integrated assessment models (Grassi et al., 2017, 2021; Popp et al., 2017). While carbon fertilization and climate change are expected to increase forest productivity globally (Kim et al., 2017), and some recent FSM studies have included climate impacts in their approaches (Favero et al., 2018; Sohngen et al., 2001; Tian et al., 2016), we do not include climate impacts in this analysis in order to provide a direct comparison with recent multi-IAM study estimates which also do not include the effects of climate change on forest productivity, and to focus on the role of timber markets and climate change mitigation policy on forest carbon.

This ForMIP assessment makes several contributions to forest and climate change mitigation literature but does have some notable limitations. First, as noted above, we do not directly address forest productivity changes under radiative forcing scenarios. Second, we do not explicitly account for the recent trends in wildfire and pest outbreaks, which could diminish forest health and carbon stocks relative to the current model parameterization. Third, while each model has methods to account for regional forest area change, not all the FSMs can explicitly account for the explicit land use the forest was converted from or to. Fourth, we do not include any national-scale FSMs with more detailed representations of localized forestry systems in our analysis, although we do present regional estimates from our global-scale models.

Results highlight the key role that existing forests play in the future global carbon balance, as well as how forest management and new tree planting are driven by both socioeconomic development and climate policy incentives. We demonstrate that economic growth and increased demand for forest biomass and land does not necessarily lead to forest carbon loss, thus global harvests and carbon storage can jointly increase with adequate incentives. We suggest that future IAM exercises should better represent forest product markets and management dynamics on existing forests, and that forest climate mitigation policies should be complemented by incentives to enhance demand for forest products and biomass.

## 2. Materials and methods

Our analysis presents results from a harmonized scenario analysis across three detailed and widely published models of the global forest sector (Table 1): the Global Timber Model (GTM): an intertemporal optimization model of global forest sector (Sedjo and Lyon, 1990; Sohngen, Mendelsohn and Sedjo, 1999; Austin et al., 2020); the Global Biosphere Management Model (GLOBIOM): a partial equilibrium model of the global land use sectors (Havlík et al., 2014; Forsell et al., 2016; Leclère et al., 2020); and the Global Forest Products Model (GFPM): a global forest product markets and timber supply simulation model (Buongiorno et al., 2003; Johnston and Radeloff, 2019).

The scenario design conforms to SSP components and forest sector pathway narratives described in (Daigneault et al., 2019), offering five alternative scenarios with varying degrees of macroeconomic and socioeconomic change (Ebi et al., 2014a, 2014b; O’Neill et al., 2014). SSP scenarios link with RCP-based emissions trajectories to simulate how forest sector adjustments can help achieve global climate targets (Box 1). Following a similar IAM multi-model assessment (Riahi et al., 2017), our scenario analysis does not account for the physical impacts of climate change, and hence the RCPs GHG emissions trajectories should be interpreted as climate mitigation pathways. Key elements of the scenario pathways include population and economic growth, demand for wood products and biomass for energy production, climate mitigation policy (via carbon prices), technological change, land use regulations, forest management intensity, and competing land rents (Table 2). All three models use the same scenario narratives and key SSP-RCP data (e.g., population, GDP, forest bioenergy demand, and carbon price) as inputs to facilitate a consistent model inter-comparison across 81 scenarios. The following sections provide additional information on our scenario design and the models used in this assessment.

### 2.1. Shared socioeconomic and climate change mitigation pathways

Global level SSPs have been developed to specify-five distinct pathways for the development of socioeconomic futures as they might unfold in absence of any explicit measures or policies to limit climate change or enhance adaptive capacity (Riahi et al., 2017; O’Neill et al., 2020). The SSPs are primarily intended to enable climate change-focused research and policy analysis, but the broad perspective and set of indicators mean that they can also be used for non-climate related scenarios such as economic and/or sustainable development (O’Neill et al., 2020). The SSPs can then be combined with RCP emissions projections to simulate actions (i.e., climate mitigation) required to meet specific global GHG trajectories. As our analysis does not account for climate change impacts, the emission trajectories can also be interpreted as climate mitigation pathways.

**Fig. 1.**
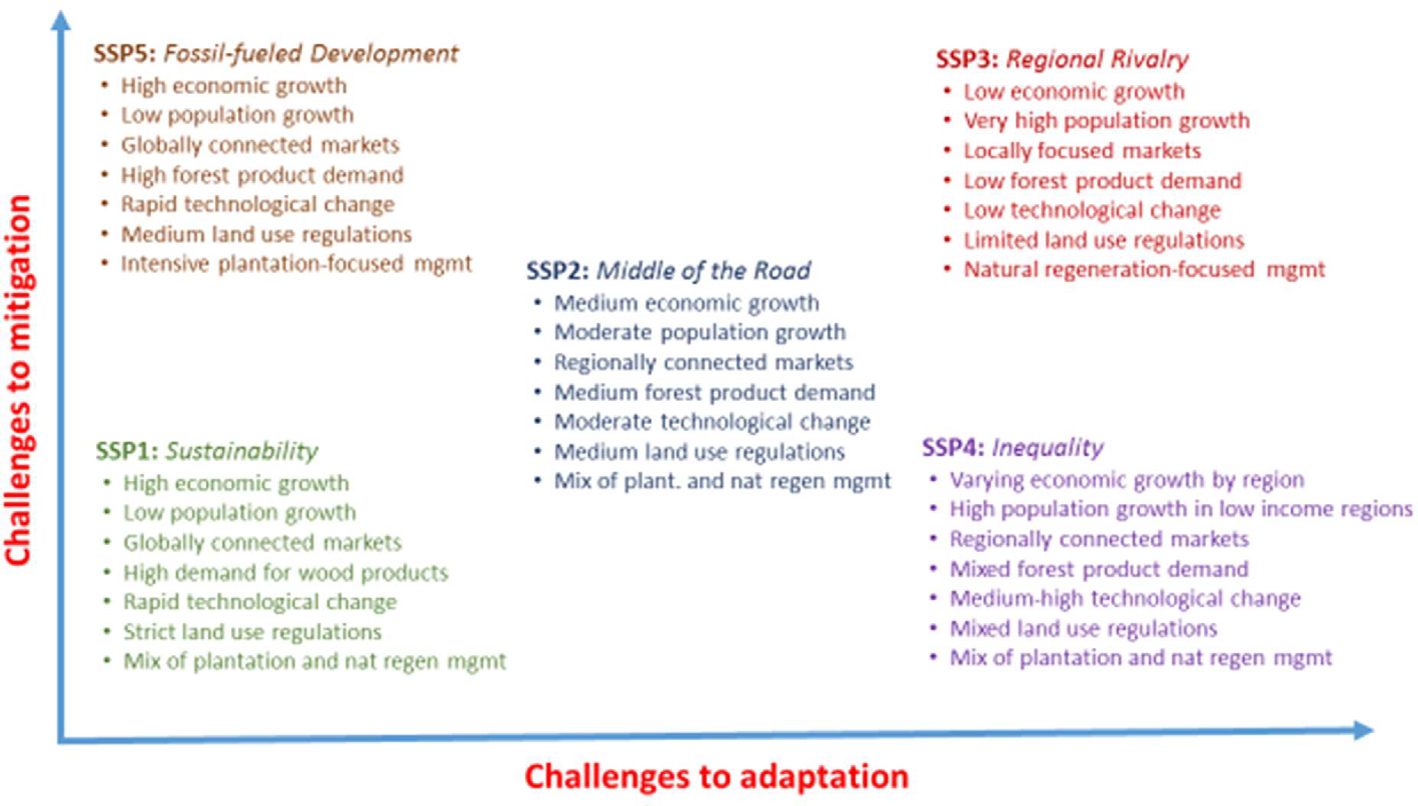
Key components of this study’s forest sector SSPs.

The SSPs were originally formulated to describe various combinations of high or low challenges to adaptation and mitigation (Ebi et al., 2014a, 2014b; O’Neill et al., 2014, 2017) (Fig. 1). The pathways range from a ‘sustainable’ world that is highly adaptive and faces relatively low socio-economic challenges (SSP1) to one that is fragmented with relatively weak global institutions and faces high population growth (SSP3). SSP4 assumes that there will be increasing inequality in global development, while SSP5 features rapid development that is driven by fossil fuels and technological change. A fifth narrative (SSP2) describes moderate challenges of both adaptation and mitigation with the intent to describe a future pathway where development trends are not extreme in any dimension and hence follow a middle-of-the road pathway relative to the other SSPs. SSP2 is often referred to as the ‘business as usual’ pathway because many indicators closely follow historical trends.

This paper builds off specific aspects of the five global SSP narratives published in the literature exploring how the global forest sector could be affected by each pathway using detailed forest sector pathway narratives in (Daigneault et al., 2019) (Fig. 1), which are outlined in section 1.1 of the supplemental materials. Key modeled forest sector elements are assumed to vary across each SSP, and include economic and population growth, international trade, technological change, wood product demand, and land use regulation (Table 2). Climate mitigation policy is introduced through the 6 RCPs (1.9–8.5 W/m2), which vary the carbon price and woody biomass demand. More details on how the FSMs incorporate these pathway elements are described below and in the supplementary material.

### 2.2. Harmonized input data

Most of the harmonized model input data was based on the IIASA SSP database (Riahi et al., 2017). Core SSP inputs included global GDP and population growth, while harmonized RCP-SSP data included carbon prices and wood-based bioenergy demand (Table S1). Carbon prices and total bioenergy demand were based on the MESSAGE-GLOBIOM estimates in the IIASA SSP database, with SSP2 values being used for missing SSP4 and SSP5 values for these two parameters (Fig. S1).^1^ The amount of woody biomass that contributed to the total bioenergy demand was based on Lauri et al. (2019), which allocate a proportion of the projected total bioenergy demand under each scenario to woody biomass, and use a constant conversion factors of 7.2 GJ/m3 wood (Fig. S2). The models were calibrated to 2015 global forest area based on UN FAO (2015). Other inputs such as biomass, timber, and carbon yields were specific to each model. All models have endogenous timber prices for wood-based commodities (sawtimber, pulpwood, etc.) and can account for forest area change and harvests from planted and natural forests, with methods varying by model.

### 2.3. Forest sector models

#### 2.3.1. Global timber model (GTM)

GTM is an economic model of forests that maximizes the net present value of consumers’ and producers’ surplus in the forestry sector. The model has been used to assess global and regional forest impacts associated with timber markets (Sedjo and Lyon, 1990), forest conservation (Sohngen, Mendelsohn and Sedjo, 1999), deforestation (Kindermann et al., 2008), climate policy (Austin et al., 2020), land use change (Tian et al., 2018), bioenergy (Favero, Daigneault and Sohngen, 2020), and climate change impacts (Favero et al., 2021). GTM’s objective function maximizes the net present value of total surplus by optimizing the age of harvesting timber and the intensity of regenerating and managing forests. GTM relies on forward-looking behavior and solves all decadal time periods at the same time over a 200-year horizon. The model accounts for nearly 300 forest types in 16 regions across the globe. Forest resources are differentiated by ecological productivity and by management and cost characteristics. The model accounts for the varying impacts of the SSPs through the adjustment of population and GDP growth, land rental rates, management costs, technological change, and consumer preferences (Table S2). Land use change is estimated using regional crop and pasture rental supply functions that differ across region and SSP based on resource scarcity and policy stringency. Carbon accounting in this version of GTM tracks stocks of aboveground biomass, harvested wood products, and harvest residuals.

#### 2.3.2. Global forest products model (GFPM)

GFPM is a recursive dynamic FSM that tracks 14 commodity groupings across 180 individual countries. The model been the main tool in recent global forest-sector outlook studies published by the US Forest Service and FAOSTAT (Buongiorno, 2012; Prestemon and Buongiorno, 2012), and has been used to assess impacts of harvested wood products accounting (Johnston and Radeloff, 2019), carbon markets (Buongiorno and Zhu, 2013, 2013), international trade policy (Buongiorno et al., 2014; Buongiorno and Johnston, 2018), and land use development (Nepal et al., 2019b). The GFPM simulates the evolution of the global forest sector by calculating successive yearly market equilibriums by maximizing a quasi-welfare function, as given by the sum of consumer and producer surpluses net of transaction costs. The model computes a market equilibrium for each periodic timestep from 2015 to 2105, subject to several economic and biophysical constraints, including a market-clearing condition which states that the sum of imports, production, and manufactured supply of a given product in each country must equal the sum of end-product consumption, exports, and demand for inputs in downstream manufacturing. GFPM equilibria were estimated based on country specific demographic and economic growth, as well as other pathway specifics for each SSP. Regional land-use change drivers were represented through an environmental-Kuznets-curve relationship with forest area. Other SSP parameters were captured within GDP and population projections and operationalized within the GFPM modeling framework through shifts in demand, supply, technological change, and transportation and shipping costs. Carbon accounting in this version of the model includes aboveground biomass stocks.

#### 2.3.3. Global Biosphere management model (GLOBIOM).

GLOBIOM is a partial equilibrium model representing land- use based activities: agriculture, forestry and bioenergy sectors (Havlík et al., 2011, 2014). The model is part of the IIASA-IAM framework and has been used since the late 2000s for various land-use and climate change mitigation scenario assessments. The model is built following a bottom-up setting based on detailed grid cell information, providing the biophysical and technical cost information. Production adjusts to meet the demand at the level of 30 economic regions. International trade representation is based on the spatial equilibrium modelling approach, where individual regions trade with each other based purely on cost competitiveness because goods are assumed to be homogenous. Market equilibrium is determined through mathematical optimization which allocates land and other resources to maximize the sum of consumer and producer surplus. The model is run recursively dynamic with a 10-year time step from 2010 to 2100. The forestry sector is represented in GLOBIOM with categories of primary products which are consumed by industrial energy, cooking fuel demand, or processed and sold on the market as final products. These products are supplied from managed forests and short rotation plantations. Harvesting cost and mean annual increments are informed by the G4M global forestry model (Kindermann et al., 2006; Kindermann et al., 2008) which in turn calculates them based on thinning strategies and length of the rotation period. The model optimizes over six land cover types: cropland, grassland, short rotation plantations, managed forests, unmanaged forests and other natural land. Economic activities are associated with the first four land cover types, and land use change is modeled with transition matrices with region and land-specific profit and conversion costs. Carbon accounting in this version of the model includes aboveground biomass stocks.

### 2.4. Scenario Analysis.

All models (n = 3) were run for each feasible RCP (n = 6) and SSP (n = 5) combination for a total of 81 scenarios (SSP3-RCP1.9, SSP3-RCP2.6, and SSP1-RCP8.5 were deemed infeasible). Estimates of forest carbon, forest area, timber harvest, and timber price were reported at the global and six region level (North America, Latin America, Europe, Former Soviet Union, Africa, Asia + Oceania). Results are largely reported as changes from 2015.

### 2.5. Model intercomparison

Each model used for this analysis has some specific parameters and assumptions (Tables S2–S4) likely to affect the results. Although significant effort was made to harmonize key data to parameterize the scenarios, the magnitude of model responses and their timing can differ given the model structure and parameters on technological change, land rents, and demand elasticity. We thus use Random Forest (Breiman, 2001) to estimate the relative importance of nine model variables, scenario parameters, and endogenous outcomes on both the aggregate and individual FSM carbon stock projections. The non-parametric method uses a series of regression trees based on repeated bootstraps of the available data and internal cross-validation for variable pruning. This machine learning approach allows for attributional analysis of factors influencing future carbon stock projections, providing insight into model-specific differences and common themes that are difficult to discern from summary figures and tables. Variable importance is estimated by identifying the number of regression trees where that variable appears and its influence on model accuracy.

## 3. Results

Our scenario estimates focus on global and regional changes between 2015 and 2105 under different socioeconomic (SSP 1–5) and climate policy (RCP 1.9–8.5) scenario combinations. The ‘baseline’ scenarios for each SSP represent the case where no climate policy is necessary to achieve a given RCP target (i.e., RCP 8.5 for SSP 2–5, RCP 6.0 for SSP1).

**Fig. 2.**
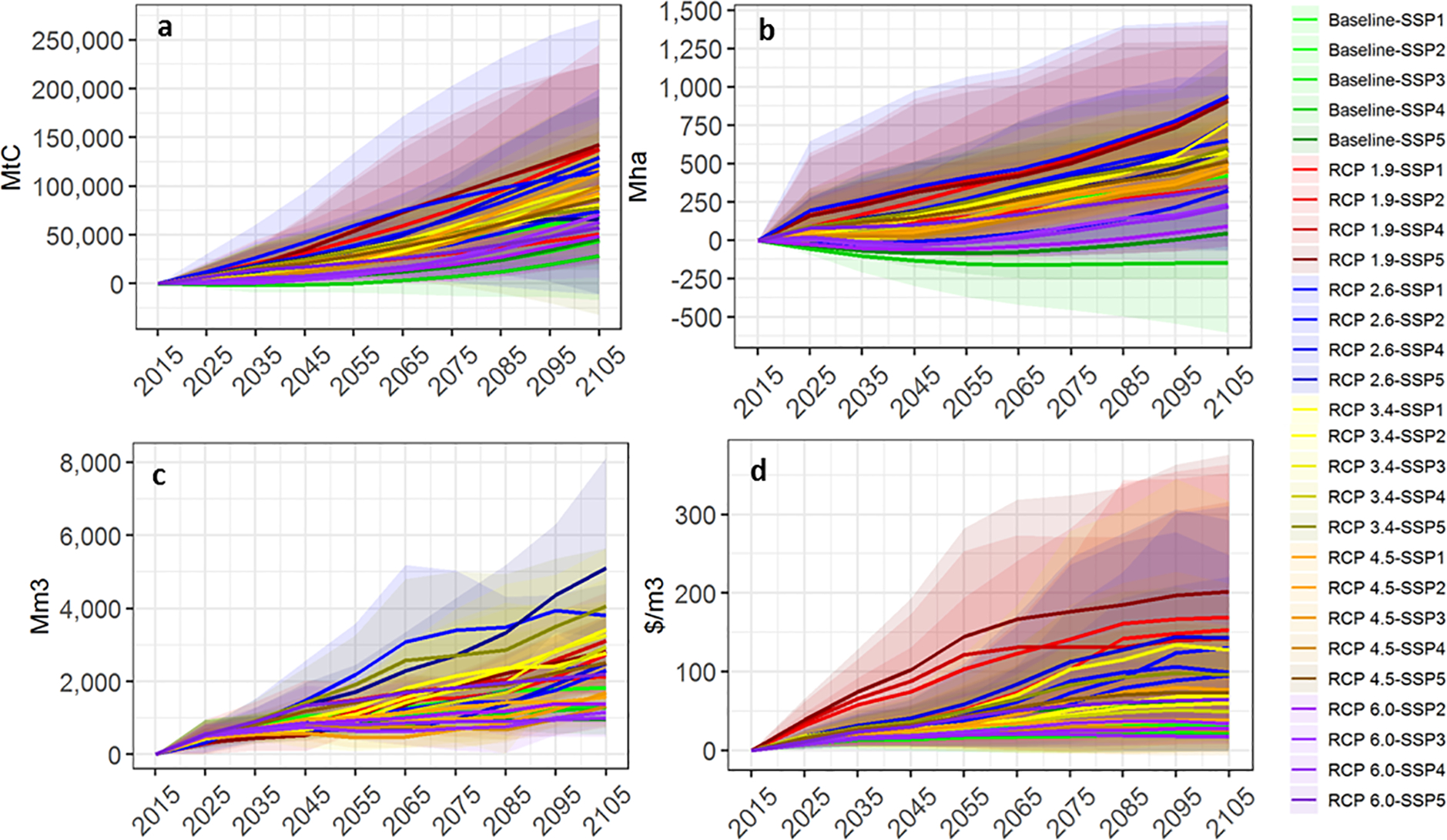
Global change in a) carbon stock (GtC), b) forest area (Mha), c) timber harvest (Mm3), and d) timber price ($/m3) from 2015 for all model pathways. Lines indicate means, and shading shows upper and lower bound of individual model estimates.

In 2015, 4.0 billion ha of global forests stored 277 GtC of aboveground carbon stock and produced 2.3 billion m3 of industrial roundwood with an average output price of $80/m3 (FAO, 2015, Table 3). Most of the 81 SSP-RCP model combinations estimate increases in forest area (85%), carbon storage (95%), wood harvests (100%), and timber prices (100%) from 2015–2105. Fig. 2 shows the range in projected model outcomes – measured as a change from 2015 – for global carbon stock (Fig. 2a), forest area (Fig. 2b), timber harvest (Fig. 2c), and timber price (Fig. 2d). Despite differences in model attributes and scenarios analyzed, Fig. 2 provides a broad perspective on directional changes in key model outputs, showing a central tendency toward increased carbon storage, forestland area, forest product output, and prices. Across all modeled scenarios, there is a greater likelihood of net forest area loss by end of century (15% of all 81 scenarios) than net carbon storage loss (5%). We elaborate on each of these key estimates in more detail below as well as provide a spreadsheet with all outputs in the supplementary data.

### 3.1. Forest area

Mean global forest area across all scenarios is projected to increase by 495 Mha from 2015 to 2105, with a range of −605 to +1435 Mha (Fig. 2b). The SSP1 and SSP5 pathways see higher levels of forest area due to relative income and productivity growth that drives resource investments and raises the opportunity costs of forest conversion. Scenarios with lower income growth and reduced trade flows (i.e., SSP3 and SSP4) combined with low or zero value for forest-based mitigation options would lead to a reduction in global forest area.

The different climate mitigation policies for the six RCPs introduce the largest variation in area. The baseline pathways result in limited expansion or loss in forest area. Our SPA climate mitigation strategies that promote biomass for energy, subsidize forest carbon sequestration and tax deforestation start with RCP 6.0 for all but the SSP1 case, and increase in stringency to RCP 1.9. The RCP 1.9-SSP5 scenario produces the largest net increase in global forest area over the next century, up nearly 1,500 Mha. For context, this 37% increase on 2015 forest area is roughly equivalent to the current forest area in the Americas. Under this scenario, carbon prices are expected to reach $1,500/tCO_2_ by 2080 and forest-based bioenergy demand more than 4.6 billion m^3^ (about 30% of total projected energy supply) while global GDP increases from $10,000/capita in 2015 to about $140,000/capita in 2105.

**Fig. 3.**
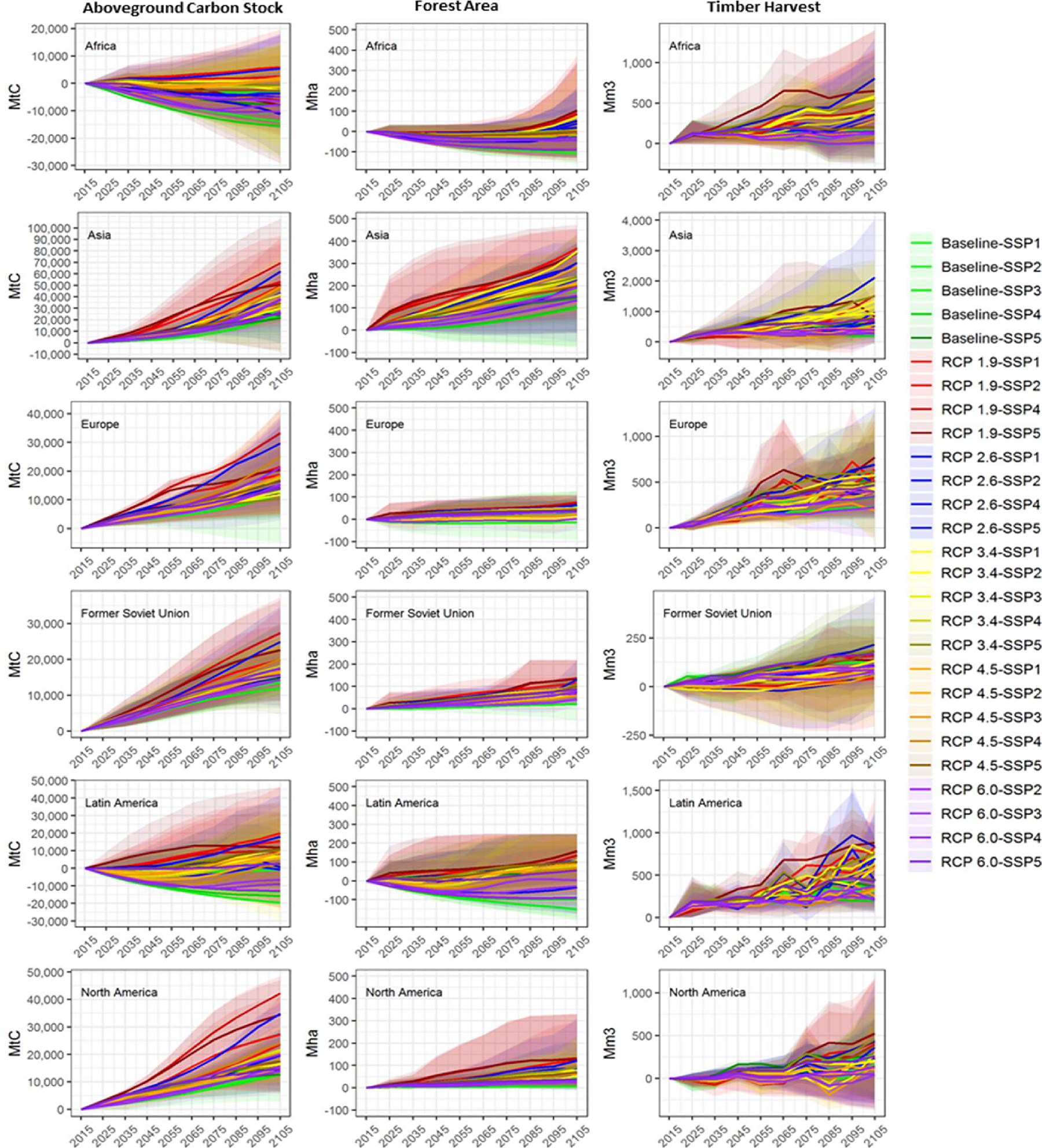
Regional change in aboveground carbon stock (GtC), forest area (Mha), and annual timber harvest (Mm3) from 2015 for all model SSP-RCP combinations. (scales vary per region).

Less stringent climate policy assumptions (i.e., higher RCP scenarios) in combination with lower income growth SSPs result in less afforestation overall. Out of the 81 runs, 12 (15%) show a possible decline in forest area. All these reductions occur under the baseline and/or the RCP 6.0 pathways, hence a combination of no to low climate policy initiatives and slower economic growth that fails to stimulate timber demand. Under the baseline-SSP3 scenario – which has the greatest forest loss – global forestland declines by an average of 144 Mha by 2105, or 3.6% below current forest area. Total forest area change by region is reported in Fig. 3.

### 3.2. Forest carbon stocks

The models project an increase in global forest carbon stocks in the future under 95% of the modeled scenarios, with an average gain of 87 GtC of forest carbon (30%) between 2015 and 2105, equivalent to 1.0 GtC/yr. Even most of the scenarios that show projected forest area loss project increased carbon stocks by 2100. The increased carbon storage is a function of afforestation, shifting harvest patterns, and management intensification. SSP4-RCP1.9 results in the largest increase in forest carbon, up 143 GtC from 2015 to 2105 (93%), or 1.6 GtC/yr. Only four model-scenario combinations result in losses of carbon stock over time: GTM’s baseline-SSP3 and GFPM’s SSP5-RCP 1.9, 2.6, and 3.4 scenarios. When averaging estimates across the three models, we find that the least optimistic scenario (Baseline-SSP3) still yields an additional 28.7 GtC (0.32 GtC/yr) by the end of the century, a 20% increase over current stocks.

Considering all model, RCP, and SSP combinations (n = 81), projected global forest carbon stocks increase by an average of 26.9 and 86.7 GtC (0.67 and 0.96 GtC/yr), respectively, by 2055 and 2105 relative to the 2015 base period (Fig. 2b), an increase of 10% by 2055 and 30% by 2105. The rate of positive change in carbon sequestration increases in the second half of the century from 0.7 GtC/yr to 1.2 GtC/yr. Regional forest C changes are relatively consistent with forest area change (Fig. 3). The greatest variability in long-term carbon stock changes are in Latin America (−30 to 46 GtC by 2105) and Asia (−8 to 108 GtC) by 2105. We also project increased carbon accumulation in the temperate and boreal regions for most scenarios. Carbon accumulation in the temperate and boreal regions results from intensified management, planting more productive timber species, and improved silviculture on existing stands.

### 3.3. Timber harvests and Prices

**Fig. 4.**
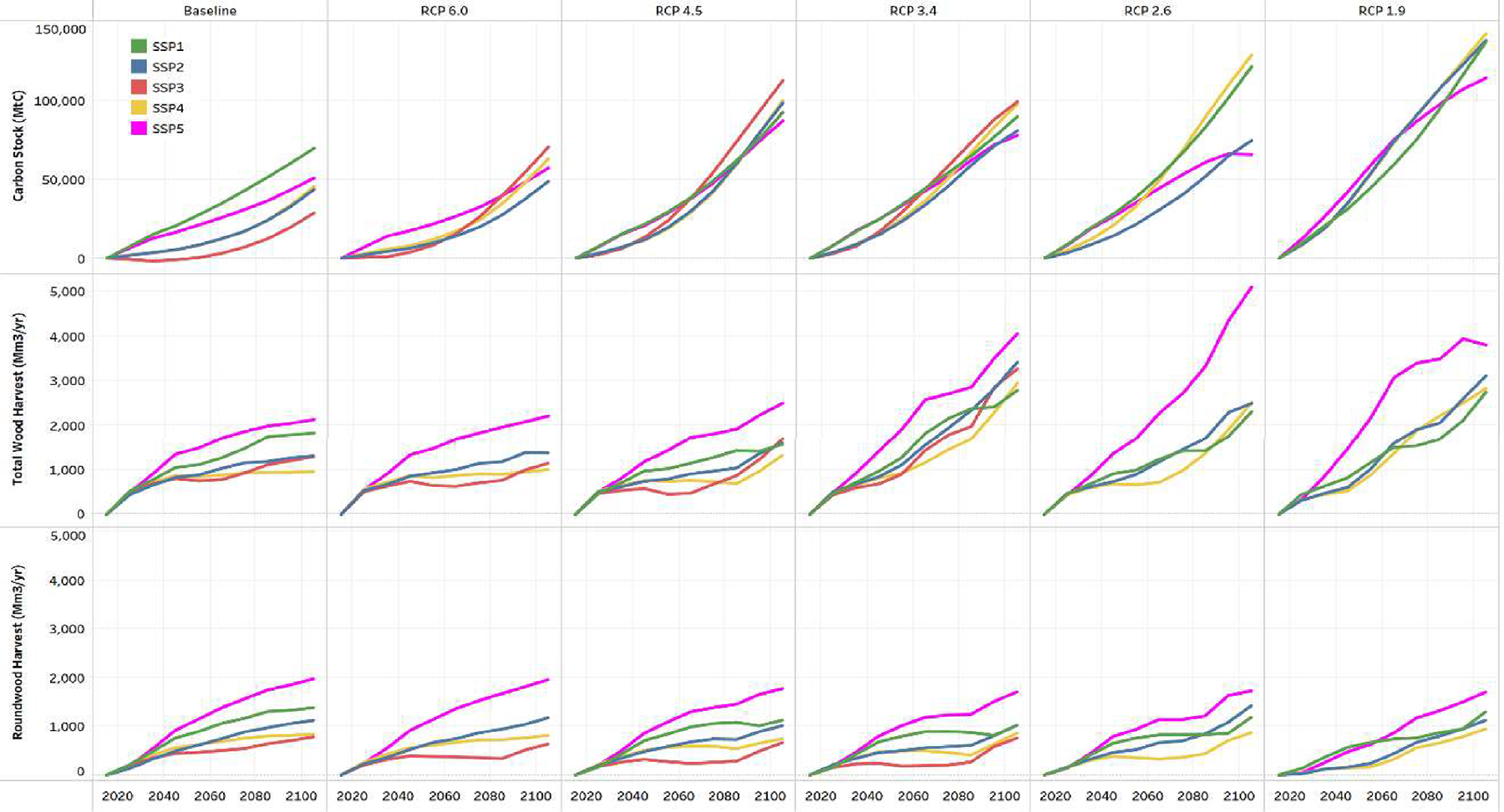
Mean change in a) global aboveground carbon stock (MtC), b) annual total wood harvest (Mm3), and c) annual industrial roundwood harvests (Mm3) from 2015 by RCP and SSP.

Global timber harvests increase by 0.5 to 8.1 billion m^3^/yr between 2015 and 2105 (Fig. 1c). SSP population and income growth trajectories shift the demand for pulpwood and sawtimber while forest bioenergy demand increases with the level of climate policy ambition. Total demand growth between 2015 and 2105 is highest under SSP5 regardless of the RCP, ranging on average from a 2.1 billion m^3^/yr increase under the baseline to a 5.1 billion m^3^/yr increase for RCP 2.6 (Fig. 4). Harvests consistently increase at lower rates for SSP4, with SSP3 following a similar trend for the base, RCP 6.0 and RCP 4.5 climate targets (1.0 – 1.6 billion m^3^/yr increase by 2105). SSP1 sees harvests increase more in RCPs 1.9 – 3.4, up by 2.3 – 2.7 billion m^3^/yr compared to 2015.

Total harvests are largest for RCPs with higher carbon prices and bioenergy requirements (RCPs 1.9–3.4), with industrial roundwood harvest levels being more consistent across RCPs, but not SSPs. This variability across SSPs indicate that socioeconomic conditions greatly affect industrial roundwood harvests, with biomass removals more heavily influenced by climate policy incentives and new market demand for wood-based bioenergy. Regionally, projected median harvests increase the most by 2105 in Latin America (440 Mm^3^/yr), Europe (466 Mm^3^/yr), and Asia (615 Mm^3^/yr) (Fig. 3). The increase in harvests are generally correlated with regional forest area expansion, particularly in the tropical regions of the globe.

Projected global timber prices, which are endogenous outcomes in each model, increase across all scenarios. Price changes are a byproduct of demand pressures, competition between timber production and preservation of existing natural forests for carbon sequestration, and long-term resource scarcity. Global timber prices are projected to increase between $17/m^3^ and $198/m^3^ over the next century (Fig. 2d). Timber prices are highly correlated with harvest volume, particularly with the more stringent climate mitigation pathways that have large increases in wood biomass demand. Projected prices increase the most under SSP5, which includes high income growth which drives demand for forest products, ranging from a $63/m^3^ real increase over the next century for the baseline to a $198/m^3^ real increase for RCP 1.9. Prices increase the least for SSPs 1 and 4, increasing from $21 to $120/m^3^ real increase by 2105, with the highest increases associated with the high biomass demand under the more stringent RCPs (2.6 and 1.9). The lower increases in timber prices for these scenarios are attributed to a combination of relatively low demand growth for both industrial roundwood and biomass.

### 3.4. Model Intercomparison

**Fig. 5.**
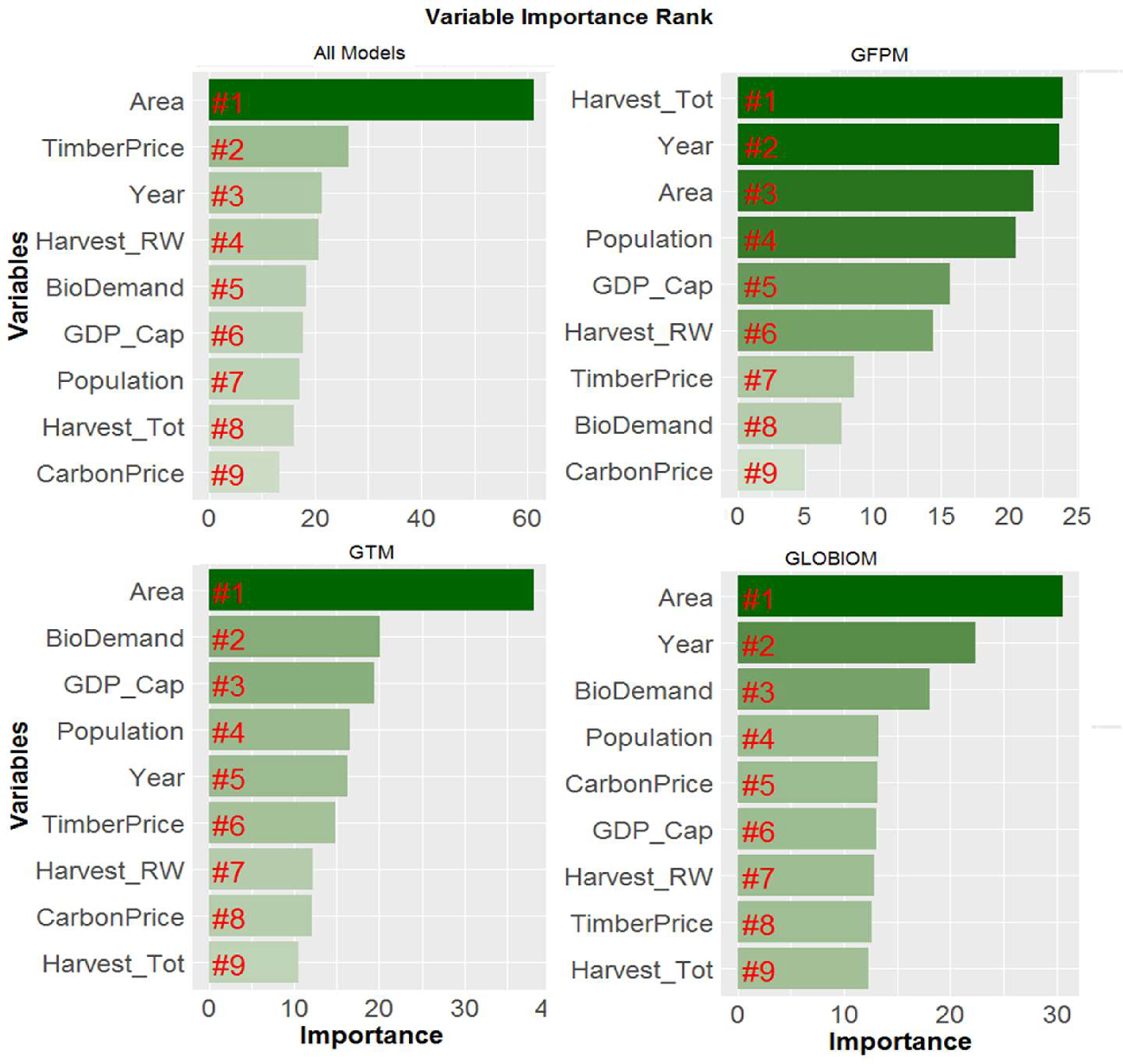
Random forest analysis of the relative importance of scenario parameters and endogenous model outcomes on projected carbon stock changes across scenarios for a) all models, b) GFPM, c) GTM, and d) GLOBIOM. [Variables: Area = forest area; TimberPrice = timber price; Year = model year; Harvest_RW = roundwood harvest; BioDemand = woody biomass demand; GDP_Cap = GDP/capita; Population = global population; Harvest_tot = roundwood + biomass harvest; CarbonPrice = carbon price].

Each forestry model used for this analysis has some specific parameters and assumptions (Tables S2–S4) likely to affect the results (Fig. 2a–d, S3). The Random Forest analysis of the three models’ variables and scenario parameters indicated that forest area has a high relative importance on forest carbon in all three models (Fig. 5) Model year was found to be important variable for GFPM and GLOBIOM as carbon stocks grow typically or decline over time in these recursive frameworks. GDP/capita and biomass demand was a strong driver of timber market demand in GTM, while the influence of model year is less important due to intertemporal optimization (some investments are made early in the simulation horizon that increase carbon stocks near team in anticipation of longer-term demand). Biomass demand has a relatively strong effect on carbon stocks in GTM and GLOBIOM but not GFPM, which is influenced more by total harvests (roundwood + biomass).

Total forest area is consistently the most important variable in determining future carbon stocks across all models. Forest area plays greater relative role in carbon storage in GLOBIOM and GTM, as land use can respond endogenously to carbon price and biomass demand drivers in these models. GTM carbon stocks are also sensitive to key demand drivers (BioDemand, GDP_Cap, Population) as this leads to both forest expansion and management intensification to boost forest productivity. In GFPM, area changes are only driven by income changes over time through the use of the Environmental Kuznets Curve method (see the SM for more documentation). CarbonPrice ranks as the factor of lowest relative importance across all models, but it is important to note that the carbon price is a key driver of forest area and management change for both GLOBIOM and GTM. Overall, these findings further highlight the uniqueness of each model framework in estimating impacts of socioeconomic and policy change on forest sector outputs.

## 4. Discussion

Our multi-FSM assessment demonstrates how widely used socioeconomic and climate policy narratives and drivers can inform global forest sector projections of industrial wood harvests, timber prices, and forest carbon stocks. The models build upon decades of analysis in the forest sector that accounts for important economic and ecological features of this sector, including ecosystem function, dynamics, trade theory, forest management, and product heterogeneity and differentiation to name a few. With exception of a few cases, these features are not included in integrated assessment and bookkeeping models which could bias those estimates (Grassi et al., 2021). We caveat that our modeled projections are not intended to be forecasts of the future, but can be interpreted as modeled projections that align with plausible future socioeconomic and policy conditions.

**Fig. 6.**
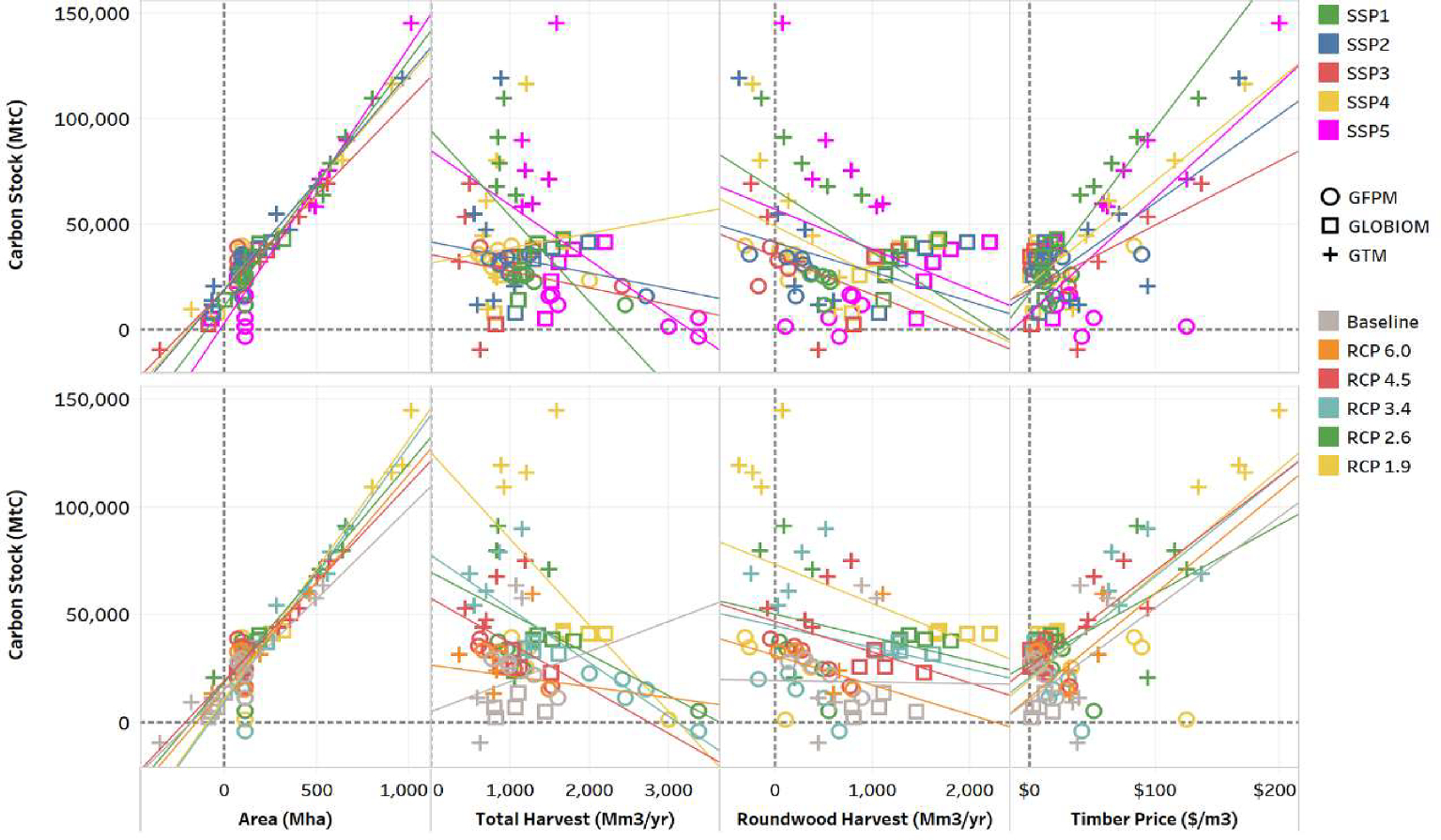
Change in global aboveground carbon stock (MtC) from 2015 relative to change in global forest area (Mha), annual wood harvest (Mm3) annual industrial roundwood harvests (Mm3) by RCP and SSP.

Overall, 95% of the scenarios indicate that forest C stocks could increase over the next 80 years, though there is a substantial variability range around these projections and all scenarios require market demand growth for forest biomass or climate policy incentives to experience this increase. The finding that forest stocks will increase because of forest sector demand in the next century is robust across several conditions and drivers, including variation in model framework, economic growth, roundwood and biomass demand, and climate and land use policy (Fig. 6). Changes in global forest C stocks are positively correlated with changes in forest area and timber price, but less so with total wood and industrial roundwood harvests. Trends in harvesting patterns, and their effects on C stocks, show substantial variation across the model frameworks. For instance, higher total harvests result in lower carbon benefits for GFPM and the opposite for GTM. The difference is largely due to how these models incorporate forest management and account for future expectations. The analysis establishes the important role that harvesting and forest management can play on the evolution of future forest stocks, which suggests that analyses that do not account for these factors may underestimate future forest carbon flows.

Our analysis builds on recent IAM assessments across SSPs and/or RCPs by explicitly representing forest management and harvest patterns on existing forests, timber markets, and carbon dynamics of forest harvest, growth, and management. Comparing our results to Popp et al. (2017) and Riahi et al. (2017), we find similar variation across SSPs and the baseline, with expected loss in forest area under the lowest growth scenarios (e.g., SSP3). However, the FSMs show more forest expansion under high growth or sustainability focused SSPs, and greater variability in forest area across models. This cross-model variation reflects differences in assumptions such as income elasticities, treatment of time dynamics, market coverage, and other important attributes that influence intensive and extensive margin responsiveness to policy drivers. We show similar trajectories for forest area to the IAM assessments across RCPs, confirming the role of forest planting and avoided deforestation in achieving climate stabilization targets. The FSMs in this study place a large portion of newly planted land into managed forest uses, while the IAMs place nearly all of it into natural forests, where there is no planned timber management or harvesting (Roe et al., 2019).

Our projected carbon stock changes span from 0.8 to 9.2 GtCO2e/yr across RCPs under SSP2 conditions through 2105 (Fig. S4). For context, Harris et al. (2021) estimate that between 2001 and 2019, global forests were a net carbon sink of 7.6 GtCO2e/yr, and (IPCC, 2021) estimates that the global land sink can add about 7 GtCO2e/yr from 2020 to 2100 under a low climate change (RCP 2.6) scenario. Further, reported average emission reductions from land use, land use change, and forestry between 2010 and 2100 for SSP2 from IAMs range from 5.1 to 9.2 GtCO2e/yr (Popp et al., 2017; Riahi et al., 2017). The larger range in FSMs results from their more explicit modeling of forest sector ecology and management activities, including harvest, growth, regrowth, and management interventions. Further, FSMs reflect regional heterogeneity in forest types and age class structure, and changes in these attributes over time, coupled with harvest and regrowth dynamics are important components of the global forest carbon cycle. IAMs, as noted above, typically model nearly all the world’s forests as unmanaged. Extensive and intensive margin interventions in the FSMs occur in response to both market and policy drivers. Forest investments under scenarios with high wood and/or carbon prices enhance forest carbon sequestration on existing forests, a result consistent with other studies (Adams et al., 1999; Galik et al., 2015; Sample, 2017; Smyth et al., 2018; Tian et al., 2018; Austin et al., 2020). These results highlight the importance of forest management decisions on current and future carbon stocks, suggesting that IAMs and other large global models should develop modeling routines that better represent timber demand and forest management when measuring carbon dynamics on forestland.

Model attributes, including the treatment of time dynamics and whether forest productivity can be endogenously increased, are important factors driving differences in projected carbon stocks across models. GTM shows the highest carbon stock gains over time across most scenarios, driven by its responsiveness to market and carbon price changes at the intensive and extensive margins. GLOBIOM and GFPM do not optimize intertemporally, so management and carbon changes in the near term are myopic of future conditions. All models show a high correlation between forest area and future carbon stocks, further validating the international policy focus on reducing forest loss and increasing afforestation. Models show variability in key outputs over time and across regions, with the largest disagreement in the directional change in future carbon stocks occurring in the tropics. Future forest model inter-comparison efforts are needed to better understand factors driving differences in regional projections, including region- or country-specific models.

While we have not modeled climate change as an additional driver in this analysis, the results suggest that forest management change driven by market and climate policy incentives has a similar effect on carbon fluxes as carbon fertilization and climate. For example, IPCC (2021) reports that the net land carbon sink from Earth System Models (ESM) will remain positive this century, with the average land sink under RCP-2.6 estimated as 6.4–9.6 Gt CO_2_e/yr, rising to 8.7–16 Gt CO_2_e/yr under RCP-8.5. Estimates from the FSM models in this paper are within the range of the RCP2.6 estimates from the ESMs, suggesting that carbon policies, combined with timber market demand and forest management will have effects of similar scale. Given the strong response of ecosystems to higher levels of atmospheric carbon in the ESMs, we suspect that if a forest carbon policy were layered on top of the climate drivers, the ecosystem response would be larger, because of the large projected increase in managed forest area that occurs when carbon in priced. While this analysis does not include climate change impacts, some of the FSM models used here have evaluated the effects in their individual modeling work. For instance, GTM has been integrated with two dynamic global vegetation models that account for predicted changes in disturbance and forest productivity by forest type and suggest that these factors have an overall positive effect on forested ecosystems globally (Favero et al., 2018; Kim et al., 2017; Tian et al., 2016). Similarly, GFPM was perturbed with carbon fertilization effects, which also found an increase in global forest stocks of 9–20% over the next century (Buongiorno, 2015). Thus, our forest carbon projections under higher forcing levels (RCP 4.5 and above) could represent a lower-bound on sequestration potential if the carbon fertilization response on forest yields is stronger than mortality or disturbance under climate change. In addition, analyses that have jointly evaluated both socioeconomic and climate impacts have almost always found that socioeconomic drivers have a much greater influence on forest sector outcomes, including carbon stocks, compared to climate impacts (Ausseil et al., 2019; Favero et al., 2018).

The broad findings of our study are generally aligned with other SSP-focused FSM assessments. With respect to changes in land area (Nepal et al., 2019b; Korhonen et al., 2021) estimated similar amounts of increases in global planted area as our study. Many FSMs estimated similar rankings of harvest volumes by SSP to our scenarios (Eriksson et al., 2020; Favero et al., 2018; Hu et al., 2018), including a threefold difference between the various SSPs, which is within the range of our global analysis (Hu, Iordan and Cherubini, 2018). Our projected increases in price changes for the RCP 1.9–3.4 scenarios – a strong driver of increased forest management and area – are similar to studies that also assume a large increase in the demand for bioenergy (Lauri et al., 2019; Favero, Daigneault and Sohngen, 2020). Similarly, studies indicate that timber prices could more than triple by the end of the century for SSP5 and increase slightly for SSP1 but remain relatively constant for the other pathways (Eriksson et al., 2020; Favero et al., 2018).

Our study results offer important insights concerning climate policy design. Specifically, while our results indicate large uncertainty ranges for key forest model outputs, general agreements on regional forest area and carbon stock trends could help policymakers prioritize regional forest planting, preservation, and management programs in climate mitigation strategies. For example, wide variability ranges in projected future carbon stocks in the tropics and a high proportion of scenarios showing reduced carbon stocks in Latin America and Africa support continued effort to reduce deforestation and forest degradation in these regions. Conversely, results suggest that strengthening forest product markets, expanding forest area, and boosting forest productivity in North America, Europe and Asia can be successful climate mitigation strategies as both timber outputs and carbon sequestration expand for these regions under most modeled scenarios. Our use of economic models provides a more realistic assessment of forest sector mitigation potential that recognizes market opportunity costs of mitigation investments, which supports tradeoff analysis of different policy designs under alternative future socioeconomic conditions (see Austin et al., 2020 for additional discussion).

While we focus on carbon stocks as our forest ecosystem service of primary interest, it is worth noting that forests provide a wide range of ecosystem services. This is especially true for natural forests, which can provide valuable biodiversity and water-related services. Carbon price policies offer some additional protection of natural forests by incentivizing reduced emissions from deforestation and degradation (Austin et al., 2020), potentially complementing the provision of other ecosystem services. However, while forest management intensification can increase carbon stocks, more intensive management and plantation expansion at the expense of naturally regenerated forests could exacerbate biodiversity concerns in some locations (Eyvindson, Repo and Mönkkönen, 2018; Leclère et al., 2020). Future forest sector model inter-comparison exercises should consider these potential tradeoffs and a wider range of ecosystem service values associated with forests.

We demonstrate key connections between forest product markets and long-term carbon storage, including the importance of complementary policies that could drive forest resource investment. Carbon accumulation and in most scenarios forest area are increased by higher timber prices (Fig. 2d) due to timber demand (industrial wood and bioenergy), and carbon policy incentives. While simulated forest carbon stocks consistently increase over time, so do harvests, which increase an average of 1.1 bil m3 by 2055 and 2.4 bil m3 by 2105 (Fig. 2c). This result suggests that it is possible to both increase forest harvest levels and forest carbon sequestration, and thus policies that incentivize forest carbon sequestration and those that stimulate demand for woody biomass for energy can be complementary (Favero, Mendelsohn and Sohngen, 2017; Baker et al., 2019).

## 5. Conclusion

We model a total of 81 future socioeconomic and climate policy scenarios across three FSMs to assess future forest climate mitigation investments and policy design. Our results demonstrate the importance of including detailed representation of the global forestry and forest market systems in mitigation analyses such as in integrated assessments of climate stabilization pathways to more accurately reflect forest market dynamics, forest management contributions to the terrestrial carbon cycle, and regional heterogeneity in forest types and policy responsiveness. Overall, we find a consistent positive trend in forest carbon stocks and timber supply through 2100, even in some scenarios with projected forest area loss, thereby highlighting the importance of carbon dynamics on existing forests and the potential gains that can be captured through forest management. Our results suggest that future IAM-based climate policy assessments should better represent forest product markets and management dynamics, and that forest climate mitigation policies should be complemented by incentives to enhance demand for forest products and biomass.

There are several limitations of this analysis that will be addressed in subsequent research efforts. Key aspects that could be expanded upon include incorporating forest productivity and ecosystem resilience impacts under the different RCPs; more explicitly accounting for land use change that results from forest conversion; and expanding ForMIP to include national- and subnational-scale forestry system models. We should also collaborate more closely with IAM community to conduct coordinated analyses that directly compare the forest-specific outcomes of mitigation and adaptation policies across model frameworks. Doing so would improve model utility of FSM analysis in general while also offering more informed recommendations on how assessments of climate stabilization and deep decarbonization can better reflect the critical role of forests, including forest management in existing systems. Subsequent analyses should also focus on regional comparison efforts and improving methods for downscaling global narratives and forest sector projections to local scales.

## Supplementary Material

Daigneault et al 2022 supplement

data tables

## Figures and Tables

**Table 1:** Key forest sector model elements.

Element	GTM	GFPM	GLOBIOM

Economic Regions	16	180	59
Resolution	regional	country	0.5°-2° grid
Sectors	Sawtimber, pulpwood, bioenergy	forest product industry	Forest industry, forestry, bioenergy, agriculture
Forest types^	302	1	6
Climate effect on forests	no	no	no
Forest products*	3	14	35
Forest products trade	n/a	Bilateral trade,	Bilateral trade, non-linear trade costs, trade-inertia constraints based on historical trade
Base year	2015	2015	2000
Calibration	Model calibrated to 2015 FAOSTAT and FRA	Model calibrated to FAOSTAT and FRA data from 2014 to 2016	Model calibrated to FAOSTAT and FRA data from 2000 to 2020
Temporal scale	10-year	5-year	10-year
Dynamics	Intertemporal	Recursive dynamic	Recursive dynamic
Biomass policy	Fixed demand	Fixed demand	Constant elasticity demand functions, which are shifted over time
Carbon policy	Carbon tax/subsidy based on carbon price applied to all pools, including HWP#	Carbon tax/subsidy based on carbon price applied to forest biomass, not for HWP	Carbon tax/subsidy based on carbon price for deforestation/ afforestation/ management, not for HWP
Endogenous response	Product price, forest area, management intensity	Product price, Timber harvest, Import, and export	Prices, quantities, land-use and management endogenous, supply side solved spatially-explicit, demand side and trade solved in regional level
Land use transition function	Agricultural land rents	Environmental Kuznets Curve	Land-use changes endogenous based on economic surplus maximization, non-linear land-use change costs, feasible areas and mapping of allowed land-use changes
Model documentation	https://u.osu.edu/forest/code-repository/	https://buongiorno.russell.wisc.edu/gfpm/	https://iiasa.github.io/GLOBIOM/index.html

^ e.g,. PNW Douglas fir, coniferous, deciduous, etc.).

* (e.g., sawlogs, pulp, etc.).

# HWP = harvested wood products

**Table 2:** Key elements for global forest sector shared socioeconomic pathways (SSPs).

Element	SSP1 (Sustainability)	SSP2 (Middle of the Road)	SSP3 (Regional Rivalry)	SSP4 (Inequality)	SSP5 (Fossil-fueled Development)

Economic growth	High	Medium	Low	HIC: High LIC: Low	High
Population Growth	Low	Medium	High	HIC: Low LIC: High	Low
Market connectivity	Global	Regional to Global	Local to Regional	HIC: Global LIC: Regional	Global
Technological change	High	Medium	Low	HIC: High LIC: Medium	High
Land use regulation	Very high	Medium	Low	HIC: High LIC: Med-low	Medium
Forest management intensity	Medium-high	Medium	Low	HIC: High LIC: Low	High
Forest product demand	Medium-high	Medium	Low	HIC: High LIC: Low	Very high

HIC: High-income countries; LIC: Low-income countries; Climate and woody biomass elements vary by RCP.

**Table 3:** Key forest sector model outputs for 2015 baseline calibration.

Metric	GTM	GFPM	GLOBIOM

Total Harvest (Mm3/yr)	1,603	2,013	1,596
Roundwood Harvest (Mm3/yr)	1,544	1,954	1,537
Biomass Harvest (Mm3/yr)	59	59	59
Forest Area (Mha)	3,960	3,997	4,033
Total Forest Non-soil C Stock (GtC)	253	287	281
Mean Roundwood Price ($/m3)	$79	$102	$55

## Box 1 Key aspects of ForMIP scenario development and model simulation

### Forest Sector Models (FSM):

3 global forest sector models that account for timber harvest, forest area, and carbon sequestration for alternative socioeconomic and climate change mitigation pathways.

### Shared Socioeconomic Pathways (SSP):

5 pathways that vary degrees of global macroeconomic and socioeconomic change, including demographics, economic growth, technological change, and policy orientations.

### Relative Concentration Pathways (RCP):

6 pathways of global greenhouse gas emissions over time. In this paper, RCPs designate global climate change mitigation targets and do not account for the physical impacts of climate change.

### Shared Policy Assumptions (SPA):

2 consistent model assumptions used to achieve climate change mitigation targets. In this paper, mitigation pathways are simulated in each FSM through specific carbon price and bioenergy demand pathways for each SSP-RCP combination.

## Footnotes

1 Although the climate mitigation pathway parameterization was limited to SSP1–3 values, we did use specific assumptions for each SSP, such that there is a unique combination of inputs for each SSP-RCP scenario.
