# Supplementary material for: How the future of the global forest sink depends on timber demand, forest management, and carbon policies
Source: Glob Environ Change. Author manuscript; Available in PMC 2023 Nov 8. (PMC10631560; doi:10.1016/j.gloenvcha.2022.102582)
Supplement: Daigneault et al 2022 supplement [file NIHMS1934424-supplement-Daigneault_et_al_2022_supplement.pdf]

Supplementary Material for:

How the future of the global forest sink depends on timber demand, forest management, and carbon policies

1. Forest Sector Shared Socioeconomic Pathways

Global level SSPs specify five distinct pathways for the development of socioeconomic futures as they might unfold in absence of any explicit measures or policies to limit climate change or enhance adaptive capacity (Riahi et al., 2017; O'Neill et al., 2017). While the specific pathways are relatively new, the concept of developing a set of alternative futures has informed global environmental assessments for decades (see Meadows et al., 1972, Gallopin et al., 1997; Nakicenovich et al., 2000). Furthermore, although the SSPs are primarily intended to enable climate change-focused research and policy analysis, the broad perspective and set of indicators mean that they can also be used for non-climate related scenarios such as economic and/or sustainable development (O'Neill et al 2014).

The pathways range from a 'sustainable' world that is highly adaptive and faces relatively low socio-economic challenges (SSP1) to one that is fragmented with relatively weak global institutions and faces high population growth (SSP3). SSP4 assumes that there will be increasing inequality in global development, while SSP5 features rapid development that is driven by fossil fuels and technological change. A fifth narrative (SSP2) describes moderate challenges of both adaptation and mitigation with the intent to describe a future pathway where development trends are not extreme in any dimension and hence follow a middle-of-the road pathway relative to the other SSPs and it is often referred to as the 'business as usual' pathway because many indicators closely follow historical trends.

This paper builds from specific aspects of the five global SSP narratives published in the literature (e.g., O'Neill et al 2014, Ebi et al 2014, O'Neill et al 2017), by expanding on how the global forest sector could be affected by each pathway (Daigneault et al., 2019). The elements that are important to the sector include economic and population growth, international trade, technological change, product demand, land use regulations, and forest management intensity and are assumed to vary across each SSP (Table S1). To isolate the socio-economic impacts from climate policy, this study also models the baseline cases for all the SSPs; that is, SSPs scenarios without a climate mitigation policy implemented.

Several components of the SSP-RCP scenarios are implemented in each forest sector model as exogenous parameters (Table S2). Most of the SSP-RCP scenario parameters are taken from SSP-database, which is publicly available through IIASA (Riahi et al., 2017). The core SSP scenario parameters included in each model are global population and GDP (Figure S1). The core RCP parameters include total bioenergy demand and carbon prices (Figure S2).

Total bioenergy is derived from a mix of woody biomass, other biomass and energy crops. Other biomass consists of agricultural residues and waste. affects forest sector by increasing woody biomass use for energy. As such, global woody biomass demand was derived from total bioenergy demand following methods in Lauri et al. (2019), using the MESSAGE-GLOBIOM model. Energy crops are woody or non-woody

biomass that is grown in dedicated energy crops plantations. [1] Total bioenergy demand is measured in terms of primary energy. Volumes of biomass are converted to energy units using factor 1 GJ=7.2 m^3 based on average density of 0.45 m^3/t and heating value 16 GJ/t (Lauri et al. 2014). Modern bioenergy consists of forest industry by-products (bark, sawdust, woodchips, black liquor, recycled wood) [2], logging residues and roundwood. All models in this analysis were calibrated to follow the respective woody biomass demand schedules for each SSP-RCP combination.

Carbon price data are not available from MESSAGE-GLOBIOM outside of SSP 1-3. While other IAMs do report carbon prices for SSP4 and SSP5, we were concerned with inconsistencies in biomass demands and other model outputs from using alternative IAMs, so we adopt the SSP2 carbon price for SSP4 and SSP5. Higher-end carbon prices faced by forest managers under these SSPs and lower emissions RCP scenarios would likely be similar since forest-based mitigation strategies are typically lower cost at the and marginal abatement costs are steep once mitigation prices exceed $200/tCO2e (Austin et al., 2020).

1.1 shared socioeconomic pathways narratives

SSP 1 - Taking the Green Road

Forest use is heavily regulated, and tropical and old growth deforestation rates are strongly reduced. Agricultural crop yields increase rapidly in low- and medium-income countries, thereby reducing the impact on forests and other natural areas through less pressure from deforestation. Forest plantation yields are also rapidly increasing across the globe because of better and more intensive management. In non-plantation forests (i.e., not plantations but potentially affected heavily by human interventions), the harvest intensity is reduced, and preservation of ecological values is emphasized. Overall consumption is decreased, and societies are characterized by low forest product consumption growth and lower resource and energy intensity. Substitution of fossil-based raw materials leads to increased use of wood in construction, and the development of novel bio-based products is rapid, while the consumption of conventional paper and paperboard decreases. Increased efficiency in the industrial wood use and new technologies permitting high recycling rates are rapidly diffused around the world and thereby reduce the demand for virgin wood for paper and board production. There is increased demand for ‘sustainably’ produced timber and non-timber forest products and forest-based amenities, with emphasis on wood sourced legally from forests under internationally recognized certification regimes.

SSP 2 – Middle of the Road

The world follows a path in which social, economic, and technological trends continue to follow historical patterns. Forest use is incompletely regulated, and tropical and old growth deforestation follow historical trajectories. Forest plantation yields increase, but at a decreasing rate, in certain parts of the globe as a result of more intensive management. Crop yields also increase to some degree, particularly in certain low- and

[1] First generation biofuels (food crops) are considered as agricultural residues and included in other biomass instead of energy crops.

[2] Recycled wood is not forest industry by-product. It is included to by-products for simplicity.

medium-income countries, but not enough to minimize the effect of the need to expand agricultural land at the expense of forests and other natural areas. Resource consumption and energy intensity increase at a decreasing rate, and as a result there is still ample demand for 'traditional' forest products, which are generally traded in regional markets. Current trends toward reduced consumption of graphics papers (newsprint, printing and writing paper) are maintained, while demand for paper-based packaging continues to expand.

SSP 3 – A Rocky Road

The world becomes increasingly compartmentalized due to national concerns about competitiveness and security. Forest use has few regulations in most parts of the world, leading to intensive harvests of timber and forest residues, alongside with continued tropical and old growth deforestation and in some developing countries even an increased deforestation relative to historic rates. Forest plantation yields improvements are minimal due to lack of investment in management and less international trade. Crop yields also decline to some degree over time, particularly in certain low- and medium-income countries, thereby leading to a significant increase in agricultural land area at the expense of forests and other natural areas. Resource and energy consumption per capita are high in developed countries, but the large population living in the developing world do not increase their consumption at the same pace. Technological improvements are halted, with little development of new biomaterials. Productivity growth is slow and focused on local solutions.

SSP 4 – A Road Divided

Highly unequal investments in human capital, combined with increasing disparities in economic opportunity and political power, lead to increasing inequalities and stratification both across and within countries. Forest use is heavily regulated in the developed world, while poor regulation in the low- and middle-income countries leads to increased degradation of forests, characterized by intensive harvesting and little attention to sustainable management or environmental consideration. Forest plantation yields and management improve in the high income countries, but the development elsewhere is minimal and limited to plantations producing raw material for the high income countries. Low crop yields in developing countries lead to a significant increase in agricultural land area, particularly near the tropics, contributing to high deforestation rates in tropical forests. Resource and energy consumption follow historical trends, with the developed world making a faster transition to lower-intensity use. Wood remains as a major fuel source in the low income countries.

SSP 5 – Taking the Highway

This world places increasing faith in competitive markets, innovation and participatory societies to produce rapid technological progress and development of human capital as the path to sustainable development. The sustainable management of forests is not consistently followed across the globe. Forest plantation yields and management increase rapidly, driven by increased demand for forest products in a globally integrated marketplace, aided by rising investments in timber growing technology. Crop yields also increase across the globe, but a strong demand for animal products continues to put pressure on converting some forest to pasture. Resource and

energy consumption grow faster than historical trends. Forest product markets are global, allowing countries to specialize and invest in new technologies and new products that are traded internationally. Packaging material and transportation fuels increase in response to market signals.

2. ForMIP Model Descriptions

2.1 Global Timber Model

This analysis uses a variant of the Global Timber Model (GTM), a dynamic optimization forest management model originally developed by Sedjo and Lyon (1990) and subsequently was updated by Sohngen et al., (1999), Daigneault et al (2012), Favero et al., (2017), and Tian et al (2018). The model relies on forward-looking behavior and solves all time periods at the same time. This "dynamic optimization" approach means that when landowners make decisions today about forest management, they do so by considering the implications of their actions today on forests in the future. For example, when forests are regenerated, the amount of money spent regenerating forests is determined consistent with future expectations about timber prices. In addition, when forests are harvested, forestland owners consider the marginal benefits and costs of waiting additional periods to harvest their trees.

In this model, sawtimber and pulpwood are drawn from the same forest resource base, which is allocated to either product after harvest. Forest resources are differentiated in several different ways, either by ecological productivity or by management and cost characteristics. To account for differences in ecological productivity, different land classes in different regions of the world will have different yield functions for timber. Data inputs used to differentiate forests by productivity are discussed below.

Furthermore, forests are broken into different types of management classes. One type is moderately valued forests (denoted by the subscript "i" below). These forests are managed in rotations and located primarily in temperate regions. A second type of management is inaccessible forest, located in regions that are costly to access. These types are denoted by the subscript "j" below. A third type is low-value forests that are lightly managed, if they are managed at all. These types are denoted by the subscript "k" in the temperate and boreal zones. These low-value lands in temperate and boreal zones are linked to inaccessible types directly, such that when inaccessible forests are harvested in boreal and temperate zones they are converted to semi-accessible forests, that is, when harvested, types in "j" convert to "k." Inaccessible forests are harvested only when the value of accessing the land exceeds the marginal access costs.

A fourth type of forests includes low-value timberland in inaccessible ("l") and semi-accessible ("m") regions of the tropical zones. Inaccessible forests in this class are harvested only when the value of accessing the land exceeds the marginal access costs. They may be converted to agriculture or returned to forestry after harvesting, depending on the opportunity costs of land and the value of future timber harvests. If the lands return to forestry, they do so in a type in m that corresponds to a similar ecological productivity level in l. The key difference between the conversions of land from inaccessible to accessible but low-value land in the temperate/boreal zones and the tropics is that lands in the temperate/boreal regions are assumed to have no opportunity

costs so they remain in forestry. In contrast, opportunity costs may be greater than 0 in the tropics and inaccessible or low-value accessible lands may convert to agriculture now or in the future.

A final type is the high-valued timber plantation ("n") type that is managed intensively. These high-value forest types can be located anywhere in the world, but at present they are principally found in subtropical regions of the United States (e.g., loblolly pine plantations), South America, southern Africa, the Iberian Peninsula, Indonesia, and Oceania including Australia and New Zealand. There are numerous types of fast-growing plantations globally with various rotation ages. Southern pines in the United States have rotation ages of approximately 30 years, while pines in other parts of the world (South America, Central America, Australia, South Africa) have rotation ages of 20 years. Eucalypts have rotation ages of around 10 years. Douglas fir has a longer rotation age, of 40 years, and teak plantations have rotations of 50 or so years. The new dedicated bioenergy plantation types in the United States are placed in this category because they are assumed to be managed similarly in 10-year rotation ages.

The model maximizes total welfare in timber markets over time across approximately 350 world timber supply regions by managing forest stand ages, compositions, management intensity, and acreage given production and land rental costs over 200 years. The supply side of the model consists of forestland with various biological yield rates that can be modified by changes in investment and management levels as well as land use changes. Superimposed on this system is a demand side that anticipates changes in demand levels for industrial sawtimber, pulpwood, and biomass though time, primarily through exogenous changes in population, per capita income, consumer preferences for wood products, and technology. The timber supply model involves the incorporation of a forward-looking forest management projections approach that is used increasingly in forestry (e.g., Sohngen et al., 1999; Adams et al., 1996). The model uses a discrete time, nonlinear, optimization approach to maximize the net present value of net surplus in timber markets.

The model's optimization problem is formally written as:

$$\max \sum_0^\infty \rho^t \left\{ \begin{array}{c} \int_0^{Q_{t,SSP}^{tot}} \left\{ D\left(Q_{t,SSP}^{ind}, Z_{t,SSP}\right) + D\left(Q_{t,SSP}^{wbio}\right) - C_{H,SSP}^{i} \left(Q_{t,SSP}^{tot}\right) \right\} dQ_{t,SSP}^{tot} - \\ \sum_i C_{G,SSP}^{i}\left(m_t^i, G_t^i\right) - \sum_i C_{N,SSP}^{i}\left(m_{t,SSP}^i, N_t^i\right) - \sum_i R_{t,SSP}^{i}\left(\sum_a X_{a,t}^{i,j,k}\right) \end{array} \right\} \quad \text{(S1)}$$

$$Q_{t,SSP}^{tot} = Q_{t,SSP}^{ind} + Q_{t,SSP}^{wbio} \quad \text{(S2)}$$

$$Q_{t,SSP}^{ind} = \pi_{SSP}^{pulp} Q_{t,SSP}^{ind} + \pi_{SSP}^{saw} Q_{t,SSP}^{ind} \quad \text{(S3)}$$

$$Q_{t,SSP}^{wbio} = \pi_{SSP}^{wbio} Q_{t,SSP}^{bio} \quad \text{(S4)}$$

where ρ^t is a discount factor, $D\left(Q_{t,SSP}^{ind}, Z_{t,SSP}\right)$ is a global demand function for industrial wood products given the quantity of wood $Q_{t,SSP}^{ind}$ and average global consumption per capita $Z_{t,SSP}$ for each *SSP*, $Q_{t,SSP}^{wbio}$ is the woody biomass demand for bioenergy production, C_H^i is the cost of harvesting and transporting timber to the mill.

Total supply is affected by several management and land costs: where C_G^i is the cost of managing G_t hectares of forest type *i* (e.g., plantation, regenerating, natural), at varying intensities *m*, C_N^i is the cost of new forestland *N* at time *t*, and $R_t^i\left(\sum_a X_{a,t}^i\right)$ is the opportunity cost of land area *X* in age class *a* at time *t*. The objective function in Eq. 1 is

nonlinear, and the model assumes that management intensity is determined at the moment of planting, and planting costs vary depending upon management intensity.

Timber demand follows the functional form $Q_{wood,t,SSP}^{ind} = A_t(Z_{t,SSP})^{\theta} P_{wood,t}^{\omega}$, where A_t is a constant, θ is income elasticity, $P_{wood,t}$ is the timber price, ω is price elasticity, and *wood* represents the type of roundwood demanded (sawtimber or pulpwood). The global demand function is for industrial roundwood, which is itself an input into products like lumber, paper, plywood, and other manufactured wood products. Total industrial demand incorporates separate demand functions for sawtimber and pulpwood. Each log harvested in the model is used proportionally in the supply of wood to sawtimber or pulpwood markets, though the proportions change endogenously over time. Demand for woody bioenergy production $Q_{t,SSP}^{wbio}$ is estimated by adjusting the total bioenergy consumption in the IIASA SSP database (Riahi et al., 2017) with the proportion of global biomass energy produced from wood by following similar assumptions in Lauri et al., (2017). Moreover, we assume different preferences for different wood products (π) according to the SSP. For example, the sustainable SSP1 scenario is likely to favor more durable timber products (sawtimber) and more sustainable bioenergy feedstocks (woody biomass) than the other SSPs. Table 2 describes the values assumed for each parameter and each SSP in the study.

GTM assumes there is an international market for timber that leads to a global market clearing price. As the price of wood for bioenergy rises to compete with industrial timber, both timber and bioenergy are traded internationally (Favero and Massetti 2014). Competition for supply equilibrates their prices.

The assumptions of each SSP impacts both the demand and supply of forest products. In particular, input costs and the rates of technological change for forest management, harvesting, and timber processing change to be in line with the future socio-economic scenarios. To account for these effects, we vary the model parameters for management intensity, forest management costs, agricultural land rental functions, and rates of technological change for harvesting and processing timber products:

$$\boldsymbol{Q_{t,SSP}^{tot}} = \sum_i \left(\sum_a \boldsymbol{H_{a,t}^i V_{a,t}^i} \left(\boldsymbol{\varphi_t^i}, \boldsymbol{m_{t0,SSP}^i} \right) \right) \quad \textbf{(S5)}$$

where the total quantity of wood depends upon the area of each age class a harvested $H_{a,t}^i$ in a given period and the yield function $V_{a,t}^i$, which is itself a function of ecological forest productivity $\varphi_t^i$and management intensity $m_{t0,SSP}^i$. Moreover, the intensity of management is chosen at the time stands are established (*t0*) and continues with the stand throughout its life. The management intensity for each SSP incorporates different assumptions.

The cost functions for harvesting and transporting roundwood and forest residues, $C_{H,SSP}^i$, are structured such that marginal costs generally increase with volume supplied to the mill or plant. Costs of managing forests, $C_{G,SSP}^i$, also follow a similar functional form. Both of these respective costs are assumed to vary by SSP ($\gamma_{t,SSP}^i$, $\beta_{t,SSP}^i$) to reflect differences in technology and efficiency over the different pathways.

Competition of land for crop and livestock is represented in the model using a land rental approach (Kim et al., 2018). The rental supply function is restricted to agricultural land that is naturally suitable for forests. It presumes that crop and pasture

land with the lowest marginal value (or economic rents) and the ability to grow forests will be converted first and that rental rates increase as more land is converted and thus becomes scarcer. We adjust the scale of the regional rental supply functions ($\alpha_{t,SSP}^{i}$) for each SSP to reflect the relative change in demand for agricultural land under the different SSPs. For example, SSP1 (sustainability) is assumed to have strict environmental and land use policies and thus would place a relatively high value on maintaining or even increasing both managed and naturally regenerating forest area. The same pathway is also expected to have high technological change across all sectors of the economy, including food production. These two factors will result in a relatively low opportunity cost for agriculture across the globe. On the contrary, SSP3 (divided) will have the opposite effect due to high population growth, low technological change, and limited land use policies.

The key components and parameters specific to GTM that are modified to represent the five SSPs are summarized in Table S3, with other assumptions listed in Table S2. The primarily demand-side components include GDP per capita, wood product preferences, and share of total bioenergy from wood. Major supply-side influences include forest management, harvest, processing costs, and shifts in annual agricultural land rents. We also adjust the forest management intensity response parameter (i.e., biomass yield increases from investment), which is used to represent technological change.

2.2 Global Forest Products Model (GFPM)

The GFPM is a recursive dynamic forest sector model that tracks 14 wood product groups across 180 individual countries. The model is calibrated to the most recent data reported by FAOSTAT by estimating input-output coefficients, and costs associated with manufacturing transportation - the GFPM solution for 2015 closely replicated the observations for the same year on production, consumption, prices, and net trade according to FAOSTAT. The GFPM is solved by calculating successive yearly market equilibriums by maximizing a quasi-welfare function, as given by the sum of consumer and producer surpluses net of transaction costs:

$$Z=\sum_{ik}\int_0^{D_{ik}} P_{ik}(D_{ik})\,dD_{ik} - \sum_{ik}\int_0^{S_{ik}} P_{ik}(S_{ik})\,dS_{ik} - \sum_{ik}\int_0^{Y_{ik}} m_{ik}(y_{ik})\,dY_{ik} - \sum_{ijk} c_{ijk}T_{ijk}$$

(S6)

where *i* and *j* refer to countries, with *k* wood product markets of price *P* as determined through end product demand *D* and wood supply *S*. The manufactured quantity of wood is denoted by *Y* at marginal cost *m*, and the quantity traded *T* at transaction cost (including tariffs) *c*. In other words, the first portion of equation (S6) is the area under the demand curve for consuming end products, while the second and third components measure the cost of production and manufacturing respectively. Finally, the last portion of equation (S6) measures the total cost of shipments. The model computes the market equilibrium subject to a number of economic and biophysical constraints, including a market clearing condition which states the sum of imports, production, and manufactured supply of a given product in a given country must equal the sum of end product consumption, exports and demand for inputs in downstream manufacturing:

$$\sum_j T_{jik} + S_{ik} + Y_{ik} = D_{ik} + \sum_n a_{ikn} Y_{in} + \sum_j T_{ijk}, \quad \text{(S7)}$$

where a_{ikn} is the input of upstream product k required in the manufacture of a given unit of downstream product n. Changes in resource efficiency are operationalized through changes in the input-output coefficients, and evolve exogenous over time according to:

$$a_{ikn,t} = a_{ikn,t-1}(1 - \eta_{ikn,t}) \quad \text{(S8)}$$

where $\Delta a_{ikn,t}$ is the periodic rate of change in input-output coefficient.
The demand in country i for final product k is assumed to follow a constant elasticity of substitution:

$$D_{ik,t} = D^*_{ik,t}\left(\frac{P_{ik,t}}{P_{ik,t-1}}\right)^{\delta_{ik}} \quad \text{(S9)}$$

where $P_{ik,t-1:;}$ is last periods price, δ''_{ik} is the price elasticity of demand for product k in region i, and current consumption at last periods price is given by:

$$D^*_{ik,t} = D_{ik,t-1}(1 + \alpha_{iy} g_{iy,t} + \alpha_{i0}) \quad \text{(S10)}$$

which is a function of last periods demand, the growth rate of GDP at time t, $g''_{I,9}$, the elasticity of demand with respect to GDP, α_{iy}, and a period trend, α_{i0}.

The cost of shipping product k from region i to region j in any given year is assumed to be a constant elasticity of substitution form:

$$c_{ijk,t} = c^*_{ijk,t}\left(\frac{T_{ik,t}}{T_{ik,t-1}}\right)^{\tau_{ik}} \quad \text{(S11)}$$

where $T_{ik,t-1}$ is last periods quantity traded, and τ_{ik} is the elasticity of transport costs with respect to quantity traded. The base period transaction cost $c_{ijk,t}$ is calibrated to estimated freight costs, observed export taxes and import ad-valorem tariffs, and endogenously determined product prices.

Supply is also described through a constant elasticity of substitution supply curve:

$$S_{ik,t} = S^*_{ik,t}\left(\frac{P_{ik,t}}{P_{ik,t-1}}\right)^{\lambda_{ik}}, \quad \text{(S12)}$$

where λ_{ik} is the price elasticity of supply for product k in region i, and current production at last periods price is given by:

$$S^*_{ik,t} = S_{ik,t-1}(1 + \beta_{iI} g^I_{it} + \beta_{ia} g^a_{it}), \quad \text{(S13)}$$

where $g^{I}_{,it}$ is the periodic rate of change of forest stock in region *i* at time *t*, $g^{a}_{,it}$ is the periodic rate of change of forest area, and β'_s indicated respective elasticities.

Land use change enters the model through changes to forest area; assumed to be a function of evolving demographics and economic growth. An environmental Kuznets curve (EKC) relationship associates changes in income per capita (Y/N) to the forest area annual growth rate, $g^{a}_{,it}$:

$$g_{it}^{a} = (\alpha_{i0} + \alpha_1 (Y/N)_{it}) e^{\alpha_2 (Y/N)_{it}}.\ \alpha_1>0 \text{ and } \alpha_2<0. \qquad \text{(S14)}$$

With parameter estimates of α_1, and α_2 estimated from historical data, and α_{i0} calibrated such that in the base year (2015) equation (S9) predicted the observed forest area growth rate, $g^{a}_{,it}$, given the observed level of income per capita, $(Y/N)_{it}$. Equation (S9) predicts negative growth rates of forest area for low income countries, which increase and become positive at higher income, and decrease progressively to zero at the highest income levels. The annual rate of change of biomass stock due to tree growth and mortality is inversely related to the forest density (residual stock level, S_{it}, per unit area, A_{it}).

SSP-RCP specific scenarios were modeled using a range of parameter assumptions, including changes in global GDP and population growth, international trade participation, resource efficiency, and wood-based bioenergy demand (Table S2). Region-specific land-use change for the different SSPs were modeled as a function of evolving demographics and economic growth represented through the EKC.

More detailed information on the model structure is provided in Buongiorno et al., (2003), including the formulations of constraints related to trade inertia, prices, manufacturing costs, transport costs, market dynamics, linear approximations of certain constraints, and annual allowable cut constraints.

2.3 Global Biosphere Model (GLOBIOM)

Global Biosphere Management Model (GLOBIOM) is a global spatially-explicit agricultural and forest sector model (Havlik et al. 2011, 2014). The forest sector representation includes forestry, forest industry and bioenergy modules (Lauri et al. 2014, 2017, 2019). The supply side of the model is solved in 0.5°-2° grid resolution while the demand and trade modelling is based on economic regions.

The model is solved recursively using biophysical data from Global Forest Model (G4M) (Kindermann et al. 2006, 2008, Gusti and Kindermann 2011) and Environmental Policy Integrated Climate Model (EPIC) (Williams 1995). Biophysical data from G4M includes biomass stocks and harvest potentials for each land use unit. Harvest potential is divided to different feedstocks (sawlogs, pulpwood, harvest loss, logging residues). G4M solves harvest potentials for GLOBIOM by assuming that all forest are normal forests. Normal forests have a uniform distribution of age-classes and in each period the oldest age-class is removed by harvesting or mortality. This is convenient from GLOBIOM recursive optimization perspective, because in normal forests harvest potentials are independent of harvest volumes and stay constant over time. Alternatively, G4M could solve harvest potential for GLOBIOM by actual age-class distribution of forests.

The model includes three forest types (primary forests, secondary forests, managed forests) and four forest management types (low intensity C/NC, high intensity C/NC). In addition to this, it is possible to exclude protected areas from production use and allocated them to primary or secondary forests. Primary forests are forestland that has not been used historically for production. Managed forests are forest land that is actively used for production while secondary forests are abandoned managed forests. Harvest volumes can be increased by increasing managed forest area (converting secondary and primary forests to managed forests) and by intensifying forest management (converting low intensity management to high intensity management).

The initial areas for different forest types are calibrated to match FRA (2020) country level data so that primary forests=FRA primary forests, managed forests =FRA production forests and secondary forests=FRA total forests-primary forests-production forests. Initial managed forest areas are allocated to low and high intensity management by using FRA planted forest data (FRA 2020) and FAOSTAT roundwood harvests data (FAO 2020). FRA planted forests are used as lower bound for high intensity management. The transition between different forest and management types is controlled by non-linear transition costs and transition constraints. Total forest area development over time is based on the SSP scenario data (IIASA 2020). Afforested areas are included into secondary forests and are not harvested under the policy assumption that these lands are planted for carbon stock preservation.

The spatial allocation of different forest and management types is based on the economic optimization, i.e., the model chooses optimal allocation of forest and management types by maximizing economic surplus given the spatially-explicit biophysical data from G4M, the country level area data from FRA and the country level biomass production data from FAOSTAT. The economic optimization typically allocates high intensity management to the most productive and easily accessible forest areas while low intensity management, primary forests and secondary forests are allocated to less productive and remote forest areas. On average, this leads a close match with the actual locations of different forest and management types. The outcome of the economic optimization can be visually assessed by using additional data on forest area use such as Nature Map Explorer (IIASA 2020b) and Word Database on Protected Areas (WDPA 2020).

The biomass demand for modern bioenergy is based on the SSP-RCP scenario data (IIASA 2020). The biomass demand for traditional bioenergy and material products are based on FAOSTAT data (FAO 2020) and shifted over time by SSP-specific GDP and population growth (IIASA 2020). Income and price elasticities for traditional bioenergy and material products are based on historical estimates, similar to Buongiorno et al. (2003) and Morland et al. (2018). Forest products bilateral trade volumes are calibrated to the BACI (Base pour I'analyse du commerce international) bilateral trade data (Gaulier and Zignago 2010) and FAOSTAT data (FAO 2020). Bilateral trade costs are based on constant elasticity functions, which are parametrized by reference volumes and costs. The trade of feedstocks and by-products is assumed to be less elastic than the trade of final products.

The forestry module includes 9 harvested products (C/NC pulpwood, C/NC sawlogs, C/NC other industrial roundwood, C/NC fuelwood, logging residues). The forest industry module includes 4 paper grades (newsprint, printing and writing papers,

packaging materials, other papers), 6 pulp grades (C/NC chemical pulp, C/NC mechanical pulp, recycled pulp, other fiber pulp), 6 mechanical forest industry products (C/NC sawnwood, C/NC plywood, C/NC fiberboard), 6 forest industry by-products (C/NC woodchips, C/NC sawdust, bark, black liquor) and 2 recycled products (recycled paper, recycled wood). The bioenergy module includes 2 final products (traditional bioenergy, modern bioenergy) and one intermediate product (wood pellets).

The model's optimization problem for forest sector is formally written as:

$$\underset{x_{ik},y_{if},y_{iho},e_{ijk},z_{imno},I_{if}}{Max} W=\sum_{ik}\int_0^{x_{ik}} D_{ik}(x_{ik})\mathrm{d}x_{ik}-\sum_{iho}c_{iho}^{tran}y_{iho}-\sum_{iho}c_{iho}^{harv}y_{iho}-\sum_{if}c_{if}^{proc}y_{if}$$

$$-\sum_{if}c_{if}^{inv}I_{if}-\sum_{ijk}\int_0^{e_{ijk}}C_{ijk}^{trade}(e_{ijk})de_{ijk}-\sum_{imn}\int_0^{z_{imn}}C_{imn}^{luc}(\sum_{o}z_{imno})dz_{imn}$$

(S15)

subject to

$$x_{ik}-\sum_{f}a_{ifk}y_{if}-\sum_{ho}a_{ihk}y_{iho}-\sum_{j}(e_{ijk}-e_{jik})\leq 0 \qquad \forall\ i,k \qquad \text{(S16)}$$

$$y_{iro}\leq\sum_{m}b_{irmo}L_{rmo} \qquad \forall\ i,r,o \qquad \text{(S17)}$$

$$y_{ilo}\leq\sum_{r}\phi_{irlo}d_{irlo}y_{iro} \qquad \forall\ i,l,o \qquad \text{(S18)}$$

$$y_{if}\leq K_{if} \qquad \forall\ i,f \qquad \text{(S19)}$$

$$K_{tif}=(1-\delta)K_{(t-1)if}+I_{tif} \qquad \forall\ i,f,t \qquad \text{(S20)}$$

$$L_{timo}=L_{(t-1)imo}+\sum_{n}z_{tinmo}-\sum_{n}z_{timno} \qquad \forall\ i,m,o,t \qquad \text{(S21)}$$

$$L_{imo}\leq\bar{L}_{imo} \qquad \forall\ i,m,o \qquad \text{(S22a)}$$

$$L_{imo}\geq\bar{L}_{imo} \qquad \forall\ i,m,o \qquad \text{(S22b)}$$

$$y_{if} \leq \sum_{k} \phi_{ifk} x_{ik} \qquad \forall\ i, f \qquad \text{(S23)}$$

where

i, j =economic regions
k= product
f=forest industry production activity
h=harvest activity
r=roundwood harvest activity $(r \subset h)$
l=logging residues harvest activity $(l \subset h)$
m,n= land-use/management types
o=land-use unit
t=time (not used if same for all variables of the equation)
W=welfare
x=consumption quantity
y=production quantity
e=trade quantity
z=area of land-use change
K=capacity
I=investments
L=land area
c^{tran} *= transport costs*
c^{proc} *= process costs*
c^{harv} *= harvest costs*
c^{inv} *= investment costs*
δ=depreciation rate
a=input-output coefficient
b=increment per area
d=biomass expansion factor
ϕ=recovery ratio
D(x) = inverse demand function
$C^{trade}(e)$ *= trade cost function*
$C^{luc}(z)$*=land-use change cost function*

Equation (S15) is the sum of consumers' and producers' surpluses. The first term of equation (S15) is the area underneath the demand curve, which represents the value of final products consumption to the consumers. The remaining terms of equation (S15) are the areas underneath the marginal cost curves, which represent the compensations paid to the producers. The second term is the transport costs of woody biomass from forest to the mill gate within each region. The third term is the harvest costs of woody biomass. The fourth term is the process costs of woody biomass. The fifth term is the investment costs.

The sixth term is the trade costs between the regions. The last term is the land-use change costs. Transport, harvest and land-use change costs are spatially-explicit, i.e., they are indexed with regions i and land-use units o. Process, investment and trade costs are not spatially-explicit, i.e., they are indexed with regions i (in case of trade costs or with import region i and export region j).

Equation (S16) is the material balance. It guarantees that products are not consumed or used as inputs in the production activities more than they are produced and traded. A production activity f uses product k as input if $a_{ifk}<0$ and produces product k as output if $a_{ifk}>0$. A harvest activity h produces just outputs, i.e., $a_{ihk}>0$.

Equations (S17) and (S18) determine the relationship between primary woody biomass supply and forest resources. Equation (S17) is the roundwood harvest constraint. This equation ensures that roundwood harvests volumes do not exceed their harvest potential for each land-use unit. The harvest potential is based on the increment and forest area data from G4M. Different forest managements are implemented in the model by assuming that harvest activities, i.e., managements, have different increments and feasible forest areas. Primary and secondary forests are not harvested, which is implemented in the model by assuming that these forest types have zero increments.

Equation (S18) is the logging residues harvest constraint. This equation connects logging residues harvest volumes to roundwood harvest volumes and limits logging residues extraction to some share of their total volume in each land-use unit. The total volume of logging residues is based on the biomass expansion factors while the share of logging residues that is allowed to be extracted on recovery ratio (Lauri et al. 2014). In the current version of the model the recovery ratio of logging residues is assumed to be 0.5 for all managements with positive increments. However, the recovery ratio of logging residues could be adapted according to management intensity and land-use units side conditions.

Equations (S19) and (S20) determine the relationship between production technologies and capital stock. Equation (S19) is the capacity constraint. Equation (S20) is capital accumulation constraint. Investments are undertaken as long as income of increasing capital stock is higher than the investment costs within each period. In the current version of the model the depreciation rate is assumed to be 0.3 in 10-year period and is same for all final products.

Equation (S21) is the land-use balance. Forestland decreases due to deforestation, i.e., changing forestland to cropland or grassland, and increases due to afforestation, i.e., changing cropland, grassland or other natural vegetation land to forestland. For sustainability reasons forestland is not allowed to be changed energy crops plantations. Within the forestland there are three forest types: primary forests, secondary forests and managed forests. For managed forests, the model chooses low intensity or high intensity management. If forest land is never used for biomass production, then it is allocated to primary forests. If the forestland is used for biomass production, then it is allocated to managed forest. If forest land is not actively use for production but has been disturbed by human activities, then it is allocated to secondary forests.

Equations (S22a) and (S22b) are additional spatially-explicit data, which is included to model to improve the outcome of economic optimization. The economic optimization typically allocates high intensity management to the most productive and easily accessible forest areas while low intensity management, primary forests and

secondary forests are allocated to less productive and remote forest areas. On average, this leads a reasonably good match with the actual locations of different forest and management types, but in single cases it might fail due to additional institutional reasons to choose alternative locations.

Equation (S23) limits recycled paper supply to a certain fraction of paper and board consumption and recycled wood supply to a certain fraction of sawnwood, plywood and fiberboard consumption.

The one period social welfare maximization problem (S15)-(S23) is first calibrated and solved for the base years 2000-2020. Then it is solved repeatedly for the desired number of periods by assuming some exogenous or model history dependent changes in the state variables. The model period is 10 years. Because most of input data is annual data, the state variables of the model are adapted to correspond to one-year periods. Because the model is solved as a social welfare maximization problem, the objective function does not include any market prices or market clearing mechanism. Market prices for products k are obtained from the shadow prices of the material balance. From programming perspective, the model is solved using the GAMS programming language and linear programming. Non-linear functions are linearized by using the piecewise-linear approximation.

The key components of GLOBIOM that are modified to represent the five SSPs are summarized in Table S2. Contrary to GTM, the effect of SSP scenarios is restricted to factors that are quantitatively documented in the SSP database (economic growth, population growth, bioenergy demand, and carbon prices).

3. Supplemental Figures and Tables

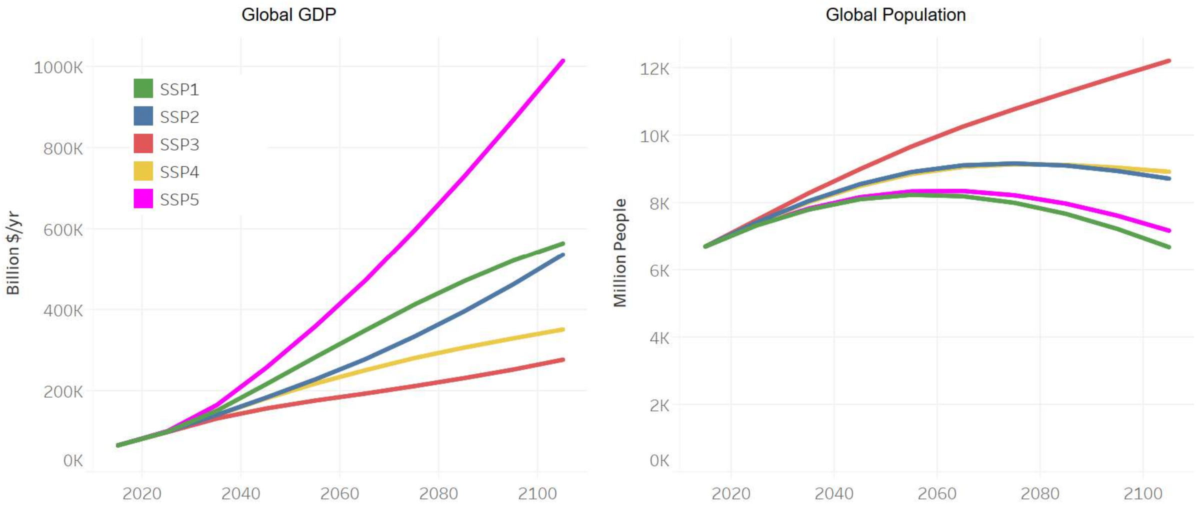

Figure S1. Global GDP and Population by SSP (Source: IIASA 2018)

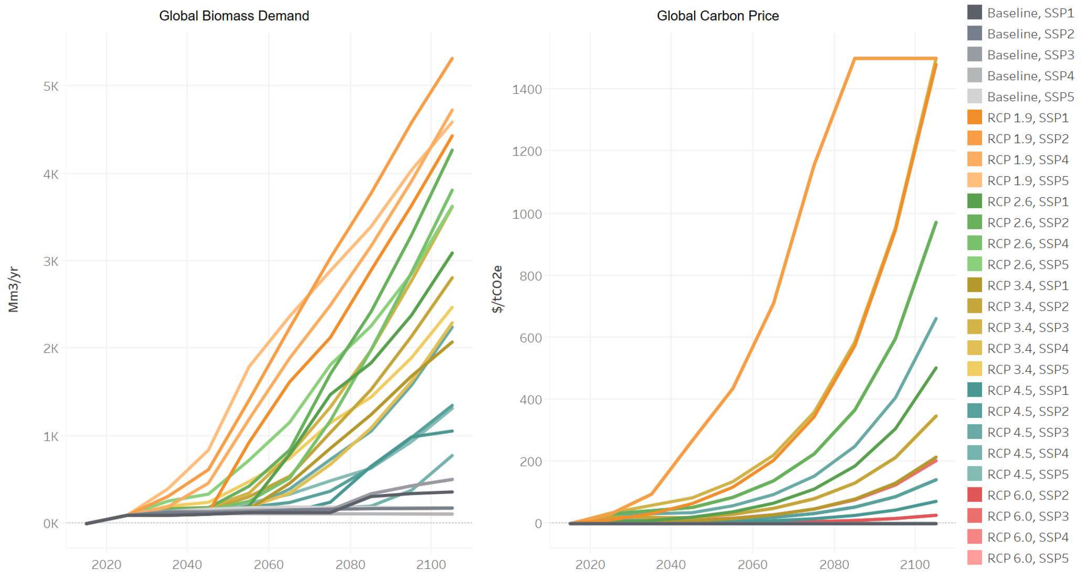

Figure S2. Total woody biomass demand and carbon prices used for SSP-RCP scenarios, as estimated by MESSAGE-GLOBIOM model (Source: IIASA 2018).

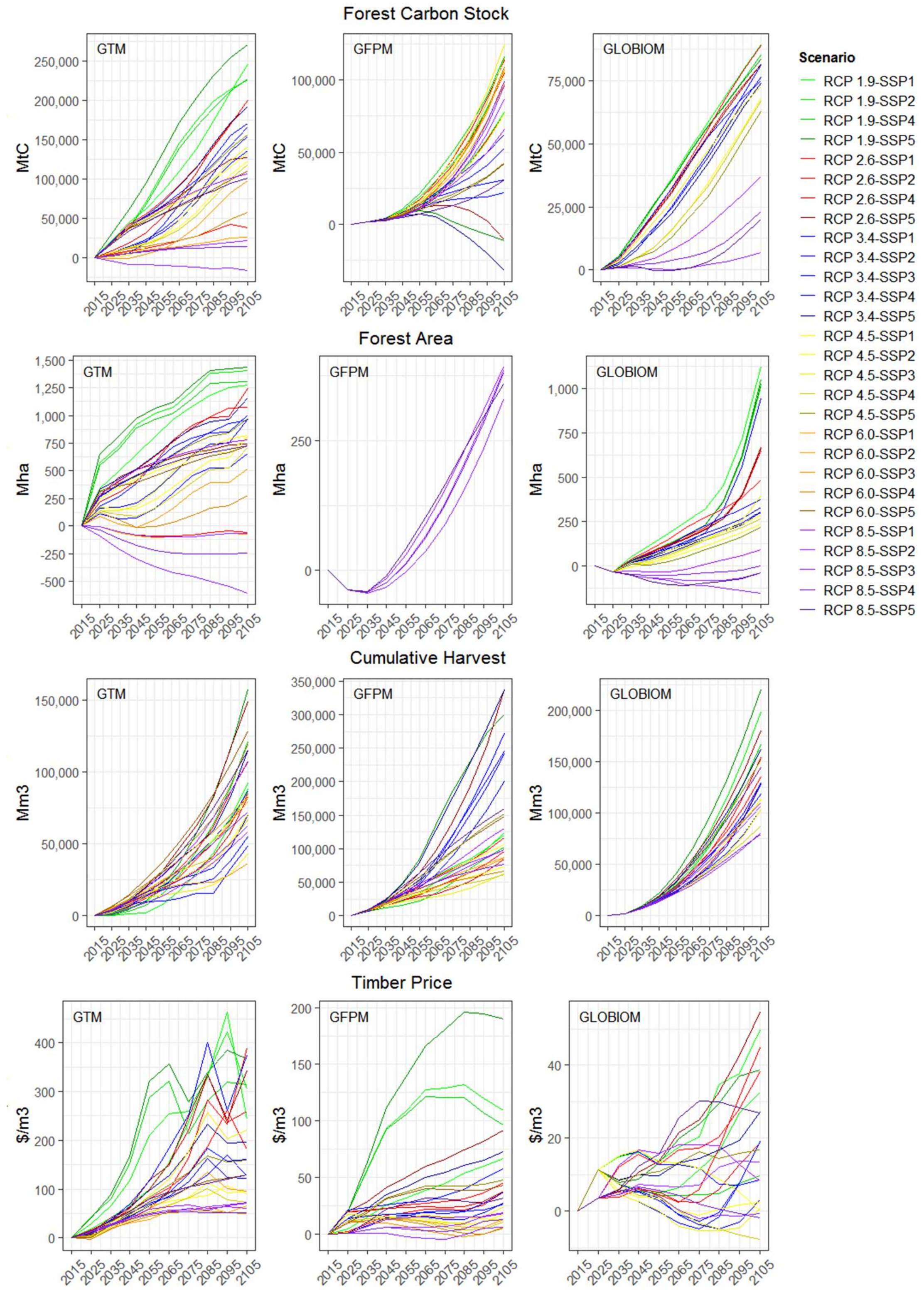

Figure S3. Comparison of global forest sector model outputs for change in global forest area, carbon, harvest, and roundwood price from 2015.

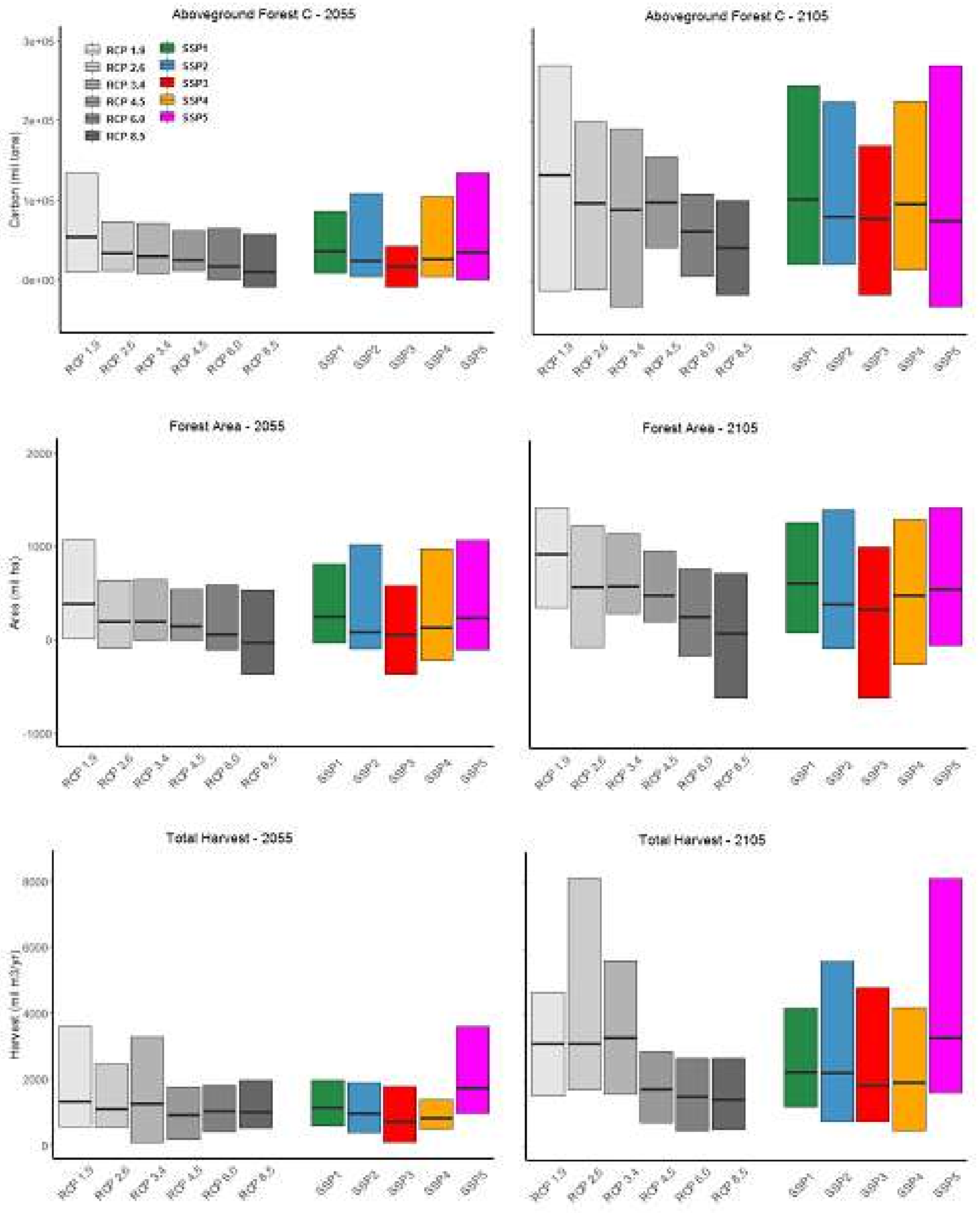

Figure S4. Mean (black bar), lower, and upper bound of changes in global forest carbon stock, forest area, and total wood harvest from 2015 by RCP and SSP.

Table S1. Source of model assumptions for SSP-RCP scenarios

Component	SSP 1	SSP 2	SSP 3	SSP 4	SSP 5
GDP	OECD GDP from SSP database				
POP	IIASA POP from SSP database				
Bioenergy demand	MESSAGE-GLOBIOM primary energy biomass from SSP database (*missing values for SSP4 and SSP5 replaced by SSP2 values*)				
Carbon price	MESSAGE-GLOBIOM carbon price from SSP database (*missing values for SSP4 and SSP5 replaced by SSP2 values*)				

Table S2. Overview of GTM model assumptions for SSP scenarios

Component	GTM Parameters	SSP 1	SSP 2	SSP 3	SSP 4	SSP 5
Global GDP per capita (annual change)	$\frac{Z_{t+1,SSP} - Z_{t,SSP}}{Z_{t,SSP}}$	OECD GDP and IIASA population from SSP database				
Wood product preference	π_{SSP}^{pulp}<br>π_{SSP}^{saw}	0.15<br>0.85	0.2<br>0.8	0.22<br>0.78	0.18<br>0.82	0.2<br>0.8
Forest management intensity response (m)	$m_{t0,SSP}^{i,j,k}$	historical +10%	historical rate	historical -10%	HIC: hist +7.5%<br>LIC: hist -7.5%	historical +7.5%
Forest management costs (% wrt t=0)<br>$C_{G,t,SSP}^{i}(\cdot) = \beta_{SSP}^{i} C_{G,t=0}^{i}(\cdot)$	β_{SSP}^{i}	90%	100%	110%	HIC: 93%<br>LIC: 110%	93%
Harvest & processing tech change (%/yr)<br>$\gamma_{SSP}^{i} = \frac{C_{H,t+1,SSP} - C_{H,t,SSP}}{C_{H,t,SSP}}$	γ_{SSP}^{i}	1.5%	0.9%	0.5%	HIC: 1.2%<br>LIC: 0.6%	1.25%
Agricultural Rents Shift (change w.r.t. to t=0)<br>$R_{t,SSP}^{i}(\cdot) = \alpha_{SSP}^{i} R_{t=0}^{i}(\cdot)$	α_{SSP}^{i}	2.0 (all expand)	1.0 (varying. change)	1.0 (all contract)	HIC: 2 (expand)<br>LIC: 1.5 (contract)	1.5 (all expand)

Note: HIC = high income countries, LIC = low income countries

4. SI References

Adams, D., Alig, R., Callaway, J., Winnett, S., McCarl, B. 1996. The Forest and Agricultural Sector Optimization Model (FASOM): Model Structure, Policy and Applications. USDA Forest Service, Pacific Northwest Experiment Station, Portland, OR.

Austin, K.G., Baker, J.S., Sohngen, B.L., Wade, C.M., Daigneault, A., Ohrel, S.B., Ragnauth, S. and Bean, A., 2020. The economic costs of planting, preserving, and managing the world's forests to mitigate climate change. *Nature Communications*, 11(1):1-9.

Bastin, J.F., Finegold, Y., Garcia, C., Mollicone, D., Rezende, M., Routh, D., Zohner, C.M. and Crowther, T.W., 2019. The global tree restoration potential. *Science*, *365*(6448), pp.76-79.

Birdsey, R., Pregitzer, K., Lucier, A. 2006. Forest carbon management in the United States. *Journal of Environmental Quality* 35(4): 1461-1469.

Buongiorno, J., Zhu, S., Zhang, D., Turner, J. and D. Tomberlin, 2003, The Global Forest Products Model, Elsevier.

Canadell, J. G., & Raupach, M. R. 2008. Managing forests for climate change mitigation. *Science* 320(5882): 1456-1457.

Coulston, John W.; Wear, David N.; Vose, James M. 2015 Complex forest dynamics indicate potential for slowing carbon accumulation in the southeastern United States. *Scientific Reports* 5: 8002. 6 p.

Cramer, W., Kicklighter, D., Bondeau, A. et al.,1999, Comparing global models of terrestrial net primary productivity (NPP): overview and key results, *Global Science Biology* 5, 1-15.

Curtis, P.G., Slay, C.M., Harris, N.L., Tyukavina, A. and Hansen, M.C., 2018. Classifying drivers of global forest loss. *Science*, *361*(6407), pp.1108-1111.

Daigneault, A. 2019. A Shared Socio-economic Pathway Approach to Assessing the Future of the New Zealand Forest Sector. *Journal of Forest Economics* (in press).

Daigneault, A., B. Sohngen, R. Sedjo 2012. Economic Approach to Assess the Forest Carbon Implications of Biomass Energy. *Environmental Science and Technology* 46 (11): 5664–71.

Daigneault. A. C. Johnston, A. Korosuo, J. Baker, N. Forsell, J. Prestemon & B. Abt. 2019. Developing Detailed Shared Socioeconomic Pathway (SSP) Narratives for the Global Forest Sector. *Journal of Forest Economics* 34: 7-45.

Ebi, K.L., Hallegatte, S., Kram, T., Arnell, N.W., Carter, T.R., Edmonds, J., Kriegler, E., Mathur, R., O'Neill, B.C., Riahi, K. and Winkler, H., 2014. A new scenario framework for climate change research: background, process, and future directions. *Climatic Change* 122(3): 363-372.

FAO, 2020, FAOSTAT database. Available at: https://www.fao.org/faostat .

FRA, 2020, Global Forest Resources Assessment, Main Report, FAO.

FAO-FRA (2015) Global Forest Resources Assessment 2015. Available at: http://www.fao.org/3/a-i4793e.pdf

Favero, A., Mendelsohn, R., & Sohngen, B. (2017). Using forests for climate mitigation: sequester carbon or produce woody biomass?. *Climatic Change*, 144(2), 195-206.

Favero, A., Mendelsohn, R., & Sohngen, B. (2018). Can the Global Forest Sector Survive 11° C Warming?. Agricultural and Resource Economics Review, 47(2), 388-413.

Forsell, N., Turkovska, O., Gusti, M., Obersteiner, M., Den Elzen, M., & Havlik, P. (2016). Assessing the INDCs' land use, land use change, and forest emission projections. *Carbon balance and management*, *11*(1), 26.

Gaulier, G. and S. Zignago, 2010, BACI: International trade database at the product level, CEPII working paper 2010-23.

Gallopin, G., Hammond, A., Raskin, P., Swart, R., 1997. *Branch Points: Global scenarios and human choice. A Report of the Global Scenario Group*. Stockholm Environment Institute 54.

Grassi, G., House, J., Dentener, F., Federici, S., den Elzen, M., & Penman, J. (2017). The key role of forests in meeting climate targets requires science for credible mitigation. *Nature Climate Change*, *7*(3), 220.

Gusti, M. and G. Kindermann, 2011, An approach to modeling land-use change and forest management on a global scale. In SIMULTECH-2011. Proc. of 1st intern. Conf. On simulation and modeling methodologies, technologies and applications, Noordwijkerhout, 180–185.
Hardie, I.W., Parks, P.J., 1997. Land use with heterogeneous land quality: an application of an area base model. *American Journal of Agricultural Economics* 79:299–310.
Havlik, P., Schneider, U., Schmid, E., et al., 2011, Global land-use implications of first and second generations biofuels targets, Energy Policy 39, 5690-5702.
Havlik, P., Valin, H., Herrero, M., et al., 2014, Climate change mitigation through livestock system transition, Proceedings of the National Academy of Science, 111, 3709-3714.
Hu, X., Iordan, C. M., & Cherubini, F. 2018. Estimating future wood outtakes in the Norwegian forestry sector under the shared socioeconomic pathways. *Global Environmental Change*, 50, 15-24.
IIASA. 2018. Shared Socioeconomic Pathway Database. http://www.iiasa.ac.at/web/home/research/researchPrograms/Energy/SSP_Scenario_Database.html.
IIASA, 2020a, SSP database. Available at: https://tntcat.iiasa.ac.at/SspDb.
IIASA, 2020b, Human impact on forest map, Nature Map Explored, https://explorer.naturemap.earth/map
Intergovernmental Panel on Climate Change (IPCC). 2013: Climate Change 2013: The Physical Science Basis. Contribution of Working Group I to the Fifth Assessment Report of the Intergovernmental Panel on Climate Change [Stocker, T.F., D. Qin, G.-K. Plattner, M. Tignor, S.K. Allen, J. Boschung, A. Nauels, Y. Xia, V. Bex and P.M. Midgley (eds.)]. Cambridge University Press, Cambridge, United Kingdom and New York, NY, USA, 1535 pp, doi:10.1017/CBO9781107415324.
Johnston, C.M. and Radeloff, V.C., 2019. Global mitigation potential of carbon stored in harvested wood products. *Proceedings of the National Academy of Sciences*, p.201904231.
Kauppi, P.E., Ausubel, J.H., Fang, J., Mather, A.S., Sedjo, R.A., Waggoner, P.E. 2006. Returning forests analyzed with the forest identity. *Proceedings of the National Academy of Sciences*. 103(46): 17574–17579, doi: 10.1073/pnas.0608343103.
Kim, S.J., Baker, J.S., Sohngen, B.L. Shell, M., 2018. Cumulative Global Forest Carbon Implications of Regional Bioenergy Expansion Policies. *Resource and Energy Economics*, 53:198-219.
Kindermann, G., Obersteiner, M., Rametsteiner, E. and I. McCallum, 2006, Predicting the deforestation-trend under different carbon-prices, Carbon Balance and Management 1, 1-17.
Kindermann, G., McCallum, I., Fritz, S. and M. Obersteiner, 2008, A global forest growing stock, biomass and carbon map based on FAO statistics, Silva Fennica 42, 387-396.
Lauri, P., Havlik, P., Kindermann, G., et al. 2014, Woody biomass energy potential in 2050, Energy Policy 66, 19-31.
Lauri, P., Forsell, N., Korosuo, A., Havlík, P., Obersteiner, M. and Nordin, A., 2017. Impact of the 2° C target on global woody biomass use. *Forest Policy and Economics*, *83*, pp.121-130.
Lauri, P., Forsell, N., Mykola, G., et al., 2019, Global woody biomass harvest volumes and forest area use under different SSP-RCP scenarios, Journal of Forest economics 34, 285-309.
Mather, A.S. 1992. The forest transition. *Area*. 24(4): 367-379.
Morland, C., Schier, F., Janzen, N., et al., 2018, Supply and demand functions for global wood markets: Specification and plausibility testing of econometric models within the global forest sector, Forest Policy and Economics 92, 92-105.
Meadows, D.H., Meadows, D.L., Randers, J., Behrens III, W.W., 1972. The Limits to Growth: a Report for the Club of Rome's Project on the Predicament of Mankind. *Universe Bookspp*. 205.
Nabuurs, G.-J., Lindner, M., Verkerk, P. J., Gunia, K., Deda, P., Michalak, R., and Grassi, G. 2013. First signs of carbon sink saturation in European forest biomass. *Nature Climate Change*. 3: 792-796. doi:10.1038/nclimate1853.

Nakicenovic, N., Alcamo, J., Grubler, A., Riahi, K., Roehrl, R. A., Rogner, H. H., & Victor, N. 2000. *Special report on emissions scenarios (SRES), a special report of Working Group III of the intergovernmental panel on climate change.* Cambridge University Press.

Nepal, P., Korhonen, J., Prestemon, J.P. and Cubbage, F.W., 2019. Projecting Global and Regional Forest Area under the Shared Socioeconomic Pathways Using an Updated Environmental Kuznets Curve Model. *Forests*, *10*(5), p.387.

O'Neill, B. C., Kriegler, E., Riahi, K., Ebi, K. L., Hallegatte, S., Carter, T. R, Mathur, R. & van Vuuren, D. P. (2014). A new scenario framework for climate change research: the concept of shared socioeconomic pathways. Climatic Change, 122(3), 387-400.

O'Neill, B.C., E. Kriegler, K.L. Ebi, E. Kemp-Benedict, K. Riahi, D.S. Rothman, B.J.van Ruijven, D.P. van Vuuren, J. Birkmann, K. Kok, M. Levy, and W. Solecki. 2017. The roads ahead. Narratives for shared socioeconomic pathways describing world futures in the 21st century. *Global Environmental Change* 42: 169-180.

Pan, Y., Birdsey, R.A., Fang, J., Houghton, R., Kauppi, P.E., Kurz, W.A., Phillips, O.L. et al., 2011. A large and persistent carbon sink in the world's forests. *Science* 333(6045): 988-993

Plantinga, A.J., Mauldin,, T., Miller., D.J. 1999. An econometric analysis of the costs of sequestering carbon in forests. *American Journal of Agricultural Economics* 81:812-24.

Popp, A., K. Calvin, S. Fujimori, P. Havlik, F. Humpenöder, E. Stehfest, B. Bodirsky, J.P. Dietrich, J. Doelmann, M. Gusti, T. Hasegawa, P. Kyle, M. Obersteiner, A. Tabeau, K. Takahashi, H. Valin, S. Waldhoff, I. Weindl, M. Wise, E. Kriegler, H. Lotze-Campen, O. Fricko, K. Riahi, D.v. Vuuren. 2017. Land use futures in the shared socio-economic pathways. *Global Environmental Change* 42: 331-335.

Riahi K., van Vuuren D. P., Kriegler E., Edmonds J., O'Neill B., Fujimori S., Bauer N., Calvin K., Dellink R., Fricko O., Lutz W., Popp A., Cuaresma J. C., Leimbach M., Kram T., Rao S., Emmerling J., Hasegawa T., Havlik P., Humpenöder F., Aleluia Da Silva L., Smith S., Stehfest E., Bosetti V., Eom J., Gernaat D., Masui T., Rogelj J., Strefler J., Drouet L., Krey V., Luderer G., Harmsen M., Takahashi K., Wise M., Baumstark L., Doelman J., Kainuma M., Klimont Z., Marangoni G., Moss R., Lotze-Campen H., Obersteiner M., Tabeau A. and Tavoni M. 2017. The Shared Socioeconomic Pathways and their Energy, Land Use, and Greenhouse Gas Emissions Implications: An Overview. *Global Environmental Change* 42:153-168.

Sedjo, R.A., and K. Lyon. 1990. *The long-term adequacy of world timber supply*. Washington, DC: Resources for the Future Press.

Sohngen, B., & Mendelsohn, R. 2003. An optimal control model of forest carbon sequestration. *American Journal of Agricultural Economics*, 85(2), 448-457.

Sohngen, B., Mendelsohn, R., Sedjo, R., 1999. Forest management, conservation, and global timber markets. *American Journal of Agricultural Economics* 81(1):1–13.

Tian, X., Sohngen, B., Baker, J., Ohrel, S. and Fawcett, A.A., 2018. Will US forests continue to be a carbon sink?.*Land Economics*, *94*(1): 97-113.

Williams, J., 1995, The Epic model. In: Singh, V. (Ed.), Water Resources Publications, 909-1000.

WDPA, 2020, World Database on Protected Areas, https://www.iucn.org/theme/protected-areas/our-work/quality-and-effectiveness/world-database-protected-areas-wdpa.
